# A fluorescent reporter and single-turnover kinetics reveal insight into BAM complex function

**DOI:** 10.1073/pnas.2514687122

**Published:** 2025-12-24

**Authors:** Whitney Nicole Bergman, Marcelo Carlos Sousa

**Affiliations:** ^a^Department of Biochemistry, University of Colorado Boulder, Boulder, CO 80309

**Keywords:** beta-barrel assembly machine, outer membrane protein folding reporter, single turnover kinetics, BAM complex

## Abstract

The β-barrel assembly machine (BAM) is an essential protein complex that folds and inserts outer membrane β-barrel proteins (OMPs) into the bacterial outer membrane. The BAM complex contains the essential BamA OMP with five soluble polypeptide transport-associated (POTRA) domains, which scaffold the essential BamD lipoprotein and the nonessential lipoproteins BamB/C/E. The importance of each BAM component has been investigated primarily using cell-based phenotypic assays, and structural data have revealed insights into the role of the BamA β-barrel in OMP folding. However, in vitro quantitative analysis for the function of each BAM component has been challenging. We describe the development of a fluorescent reporter of OMP folding, bOmpA-A488, which we use to obtain single-turnover kinetic parameters for wild-type BAM complex in vitro. We observe a k_fold_ of 0.78 ± 0.15 min^−1^ and approximate substrate affinity of 3.1 ± 1.1 µM consistent with estimates of in vivo requirements. Furthermore, we find that while BamA alone is inactive in *Escherichia coli* lipid liposomes, BamAB and BamAD subcomplexes have activities similar to that of the holoBAM complex. This suggests that OMP folding and insertion is catalyzed by BamA while the accessory lipoproteins maintain BamA in a catalytically competent conformation. We also find that, contrary to prevailing models, POTRA domain deletions that include POTRA3, which is essential in cells, do not drastically impact activity. This indicates that the first three POTRA domains do not play a major role in binding or folding OMPs under single-turnover conditions, suggesting a different role in cells.

## Significance

The folding of outer membrane proteins (OMPs) in Gram-negative bacteria requires the essential β-barrel assembly machine (BAM). By developing a fluorescent OMP folding reporter, we have unlocked insight into BAM activity in vitro, opening the door for rigorous evaluation of BAM mutants and putative inhibitors. Furthermore, we found that BamA alone is inactive, but its activity can be rescued by either the essential BamD or the nonessential BamB, suggesting partially redundant roles in stabilizing active BamA conformations. We also found that, contrary to current models, the BamA POTRA1-3 domains do not contribute significantly to catalysis, despite being essential for bacterial growth. Thus, our quantitative approach allows for robust testing of mechanistic BAM models.

Gram-negative bacteria comprise a clinically important subset of bacteria that possess a unique cell envelope; they have two membranes, which are separated by the cell wall-containing periplasm (1). The outer membrane serves a vital function for these bacteria by acting as a selectively permeable barrier, protecting the cell from antibiotics and lipophilic agents (2). Additionally, outer membrane proteins (OMPs) with typical transmembrane (TM) β-barrels facilitate important cellular processes such as nutrient transport and drug efflux (3). While the outer membrane presents a roadblock for antibiotic use, an effective antimicrobial strategy might lie in targeting key components of OMP biogenesis (4).

OMPs are ribosomally synthesized in the cytosol and then exported to the periplasm by the Sec translocon of the inner membrane (5–7). To prevent aggregation, OMPs can be bound by periplasmic chaperones SurA, Skp, and DegP (8–14). In the currently prevailing model, OMPs are shuttled by SurA to the β-barrel assembly machine (BAM), which folds and inserts the OMP into the outer membrane (15, 16). BAM function is essential for Gram-negative survival (16, 17).

The prototypical *Escherichia coli* BAM complex consists of the β-barrel OMP BamA and the lipoproteins BamBCDE (16–19). Cell growth and membrane permeability assays have revealed important insight into the effects of BAM component deletion. BamA and BamD are both conserved and essential components in Gram-negatives as depletion of either is lethal (16, 17). Individual deletion of BamB, BamC, or BamE is tolerated, suggesting these play less vital roles in OMP biogenesis (16, 18, 20). BamA has two structurally distinct domains: the TM β-barrel domain and the soluble periplasmic polypeptide transport-associated (POTRA) domains, containing 5 repeats in *E. coli* (21–25). BamCDE bind BamA through POTRA 5 and BamB binds BamA through POTRA 3 (18, 24, 26–28). The POTRAs and BamB have also been identified as SurA docking sites (29–32). Intriguingly, POTRA domains 3 to 5 are essential while deletion of POTRAs1-2 is tolerated in *E. coli* (24). Accordingly, the POTRA domains have been proposed to bind substrate OMP and are suggested to facilitate the formation of β-strands in the OMP substrate (22, 24, 33, 34). However, it is still unclear how these growth phenotypes are related to the OMP folding mechanism of BAM, due, at least in part, to limitations in the available in vitro folding assays.

The BAM complex activity can be studied with in vitro OMP folding assays, which typically rely on a characteristic “heat modifiability” of OMPs (35) that allows the separation and quantification of folded and unfolded OMP species with SDS-polyacrylamide gel electrophoresis (SDS-PAGE). While accessible, the assay is discontinuous, requires effective folding quenching and substantial postprocessing, and is difficult to implement for fast reactions. An alternative assay uses the OMP protease OmpT as a BAM substrate. As OmpT folds, it cleaves a quenched fluorescent peptide resulting in fluorescence increase (36–41). However, it is difficult to extract BAM kinetic parameters from this assay.

Here, we report the development of a platform to quantitatively measure the activity of the BAM complex in vitro. We first demonstrate that a fluorescently labeled OMP increases fluorescence when folded into membranes, allowing for folding rates to be quantitatively tracked continuously with high sensitivity and dynamic range. We then use this fluorescent OMP substrate reporter under single-turnover conditions to report kinetic parameters for wild-type BAM complex as well as mutants with deletions of POTRA domains and lipoproteins yielding insight into BAM function. The results highlight the potential for this tool in understanding the molecular mechanism of the BAM complex and the evaluation of inhibitors.

## Results

### Development of an OMP Folding Reporter.

**Fig. 1.**
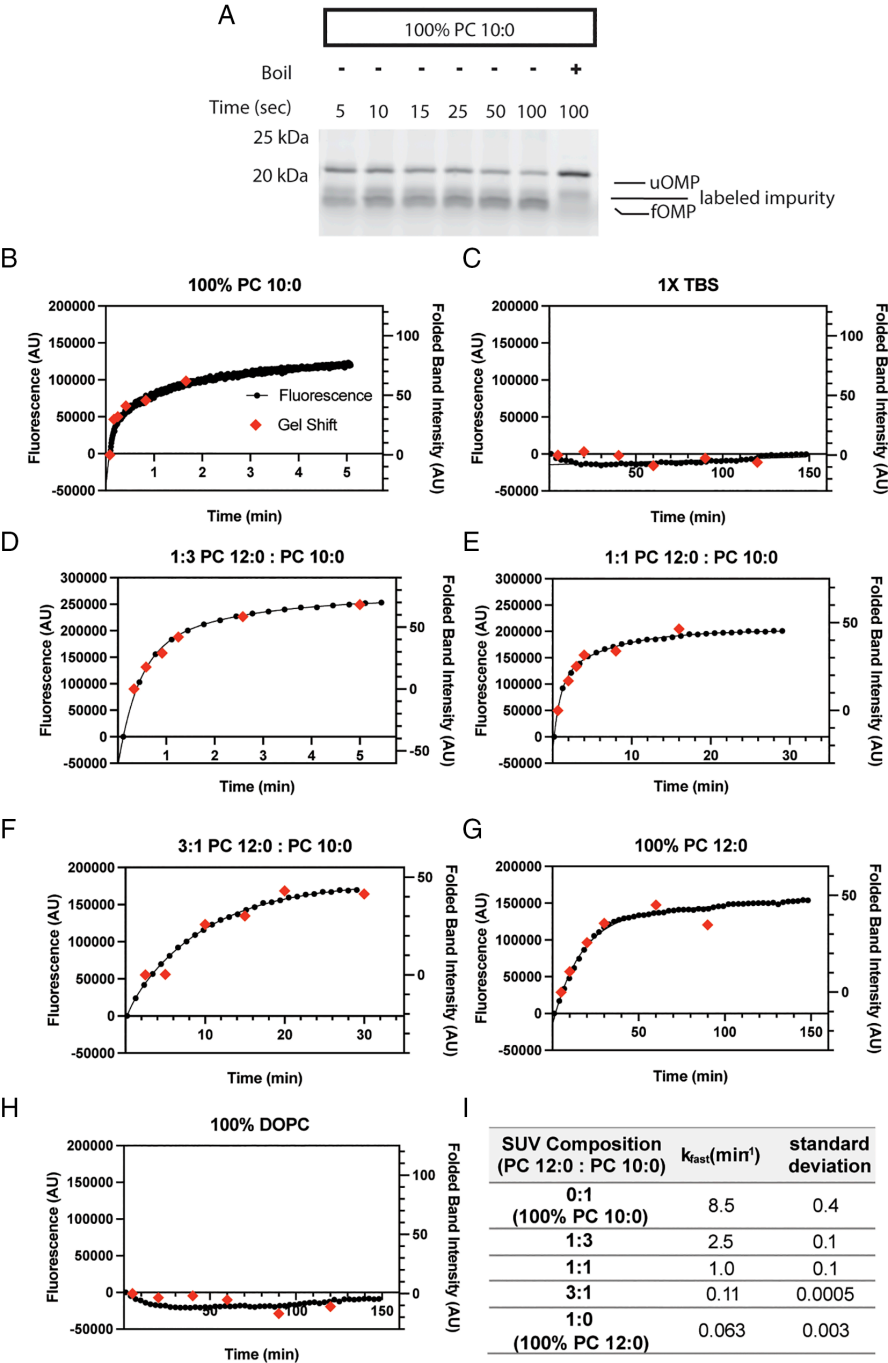
bOmpA-A488 folding monitored by gel-shift and fluorescence. (*A*) Representative SDS-PAGE heat modifiability assay for bOmpA-A488 folding into PC 10:0 liposomes. Indicated are unfolded (uOMP) and folded (fOMP) bOmpA-A488 and a labeled impurity present in all conditions. (*B*–*H*) Representative bulk fluorescence (left axis, black dots) vs. time of bOmpA-A488 in solutions of mixed PC liposomes (*B* and *D*–*G*), buffer (*C*), or DOPC liposomes (*H*), overlaid with data from folding quantification from SDS-PAGE (right axis, red diamonds), (*I*) All fluorescence data were fitted to double exponential functions, except the 3:1 PC 12:0: PC 10:0 condition, which was fitted to a single exponential, and the fast rate constant is reported as the average of two independent replicates ± STD.

The folding status of OMPs is often evaluated using SDS-PAGE, taking advantage of their characteristic “heat modifiability” (35). In this approach, when OMPs are solubilized and boiled, they denature, resulting in a single denatured band in the gels. However, SDS solubilization without boiling does not denature folded OMPs that run with distinct mobility in SDS-PAGE, allowing quantification of the folded fraction. We sought to develop a fluorescent reporter to assay OMP folding without the need for SDS-PAGE to separate folded and unfolded species. We settled on the TM β-barrel of OmpA (amino acids 22-197 with an N-terminal methionine, bOmpA), a construct that has been previously shown to spontaneously fold into liposomes composed of short chain Phosphatidyl-Choline (PC) lipids (12, 42–50). A cysteine was introduced between residues 2 and 3 of the bOmpA construct, just outside the TM region, and was labeled with Alexa Fluor 488-maleimide (herein referred to bOmpA-A488). A folding assay was initiated by mixing urea-denatured bOmpA-A488 with PC 10:0 liposomes followed by monitoring of fluorescence at 516 nm. We observed a progressive, saturable increase in fluorescence over time (Fig. 1*B*, black dots). Samples were also taken from the folding reaction at different time points and evaluated for folding using the standard SDS-PAGE based “heat modifiability” assay (Fig. 1*A*). There was an excellent correlation between the rate of fluorescence increase and the increase in intensity of the folded bOmpA-A488 band over time (Fig. 1*B*, red diamonds). The change in fluorescence over time was best fit to a double exponential, consistent with previous reports of OMP folding in these lipids (44, 50, 51), which yielded a fast folding rate constant k_fast_ of 8.5 ± 0.4 min^−1^ (mean ± STD from two independent reactions) (Fig. 1*I*). As a control, mixing of urea-denatured bOmpA-A488 with buffer without liposomes resulted in negligible fluorescence changes and no folding of bOmpA-A488 (Fig. 1*C*).

The phospholipid packing order in liposomes increases with the length of the phospholipid acyl chains and decreases the rate of spontaneous OMP folding (51, 52). We thus tested the ability of bOmpA-A488 to report the rate of folding in liposomes with increasing ratios of PC 12:0/PC 10:0 phospholipids. As expected, increased PC 12:0 lipid fraction resulted in decreased rates of bOmpA-A488 fluorescence (Fig. 1 *D*–*G*, black dots). In all cases, fluorescence change showed excellent correlation with the increase in intensity of the folded bOmpA-A488 band over time in the standard “heat modifiability” assay (Fig. 1 *D*–*G*, red diamonds). Double exponential fits yielded k_fast_ values of 2.5 ± 0.1, 1.0 ± 0.1, 0.11 ± 0.0005, 0.063 ± 0.003 min^−1^ (mean ± STD from two independent reactions) for PC 12:0-to-PC 10:0 ratios of 1:3, 1:1, 3:1, and 1:0 (100% PC 12:0), respectively (Fig. 1*I*). Furthermore, we tested folding of bOmpA-A488 into liposomes composed of longer PC 18:1(∆9-cis) phospholipids (DOPC), which do not allow spontaneous OMP folding (51). As expected, negligible fluorescence changes and no folding of bOmpA-A488 were observed in the time frame of the experiment in these liposomes (Fig. 1*H*). Taken together, these results indicate that bOmpA-A488 is a suitable reporter to evaluate folding by direct monitoring of fluorescence changes at 516 nm.

### Single-Turnover Kinetics of the BAM Complex Reveals the Rate of Folding Catalysis and Estimates of Substrate Affinity.

**Fig. 2.**
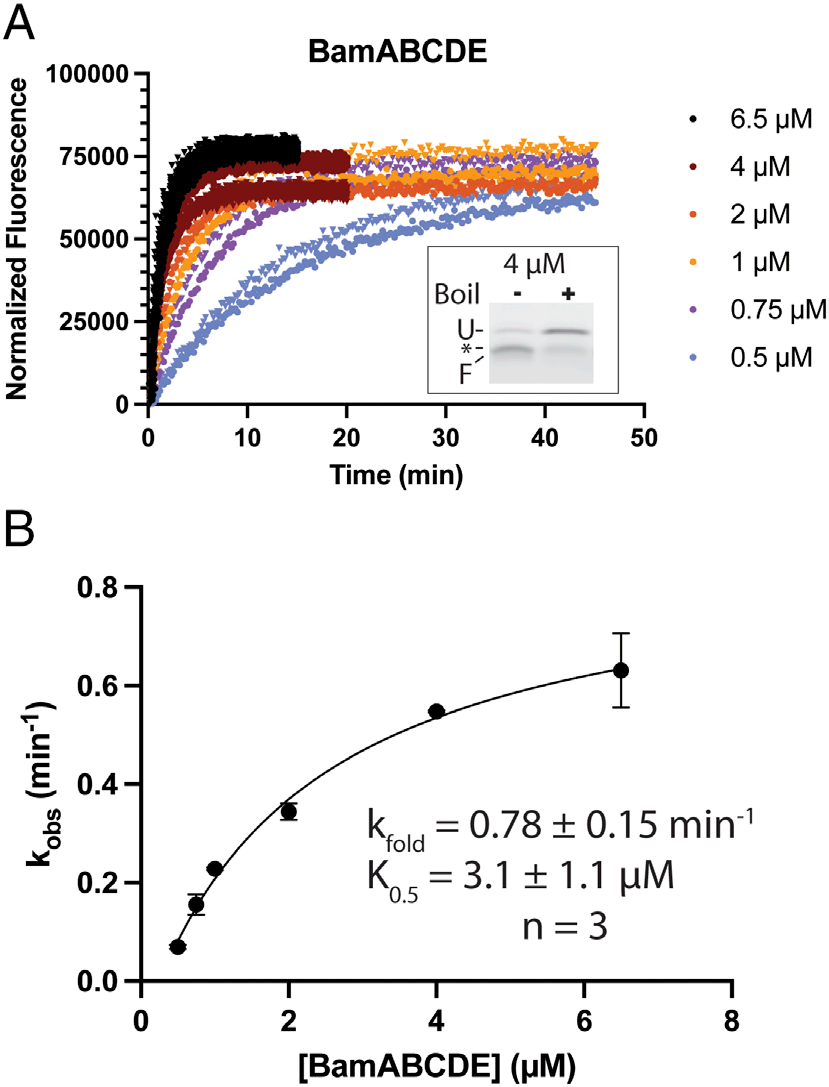
Single-turnover kinetics of wild-type BamABCDE complex. (*A*) Representative fluorescence vs. time data from a BamABCDE activity assay. Background-corrected data were fitted to a single exponential function and normalized to the Y-intercept value. Technical replicates for given concentrations are shown as triangles of the same color. *Inset*: representative heat modifiability assay for bOmpA-A488 with 4 µM BamABCDE after 20 min. Indicated are unfolded (U) and folded (F) bOmpA-A488, and a fluorescent contaminant (asterisk). (*B*) Single exponential rate constants from (*A*) were plotted against BamABCDE concentration and fitted to an “offset” hyperbolic curve (Eq. **1**). Data points are the mean of two technical replicates, with STD error bars. *Inset*: fit parameters for three independent biological replicates, reported as the mean ±STD.

We next used the bOmpA-A488 reporter to quantify folding catalysis by BAM. Wild-type BAM complex (BamABCDE) was reconstituted into liposomes composed of *E. coli* polar lipids (EPL) using a previously described dialysis method (38, 49). Standard folding assays were carried out by mixing urea-denatured bOmpA-A488 with the BamABCDE proteoliposomes and the periplasmic chaperone SurA (38, 46, 49). Due to OMP aggregation propensity and a potential for limited capacity of OMP folding within highly packed proteoliposomes, we chose to utilize single-turnover conditions to probe BAM catalysis where [bOmpA-A488] << [BAM]. To verify such conditions, we tested bOmpA-A488 concentrations ranging from 2 to 50 nM while keeping the BAM concentration at 0.75 µM. We observed exponential increases in fluorescence that scaled with the bOmpA-A488 concentration but resulted in indistinguishable exponential folding rates, confirming single-turnover conditions (*SI Appendix*, Fig. S1). Next, we carried out folding assays at 10 nM bOmpA-A488 and increasing concentrations of BamABCDE proteoliposomes in the µM range that resulted in exponential fluorescence increases that saturated at rates proportional to the concentration of BAM (Fig. 2*A*). Reaction endpoints confirmed that the fluorescence increases correlated with bOmpA-A488 folding (Fig. 2 *A*, *Inset*). The single exponential folding rate constants obtained from the fluorescence curves in Fig. 2*A* were plotted against the concentration of BAM to yield a hyperbolic curve that was fitted to an x-axis “offset” square hyperbola (Eq. **1**, see *Materials and Methods*). The asymptote reflects the intrinsic rate of BAM catalysis (k_fold_) observable at full substrate saturation, while the concentration of BAM at half the k_fold_ represents the constant value, K_0.5_, composed of the substrate association (k_1_) and dissociation (k_-1_) rate constants, and k_fold_ (Scheme **1** and Eq. **2**). We report that wild-type BamABCDE complex has a k_fold_ of 0.78 ± 0.15 min^−1^ and K_0.5_ of 3.1 ± 1.1 µM (mean ± STD from three independent BAM reconstitutions). Assuming the 0.78 ± 0.15 min^−1^ k_fold_ is slow compared to the substrate dissociation constant k_-1_, the K_0.5_ can serve as an approximation of the substrate dissociation constant (K_d_). These parameters were highly reproducible across separate BAM protein preparations and reconstitutions. Importantly, we saw no folding of bOmpA-A488 into empty EPL liposomes, confirming that the folding we observed is BAM-catalyzed (*SI Appendix*, Fig. S2). Furthermore, the k_fold_ we observe is predicted to support normal doubling times in *E. coli* (53).

### BamA by Itself Is Defective at Catalyzing Folding in *E. coli* Lipid Membranes and the E470K Mutation Does Not Rescue Activity.

**Fig. 3.**
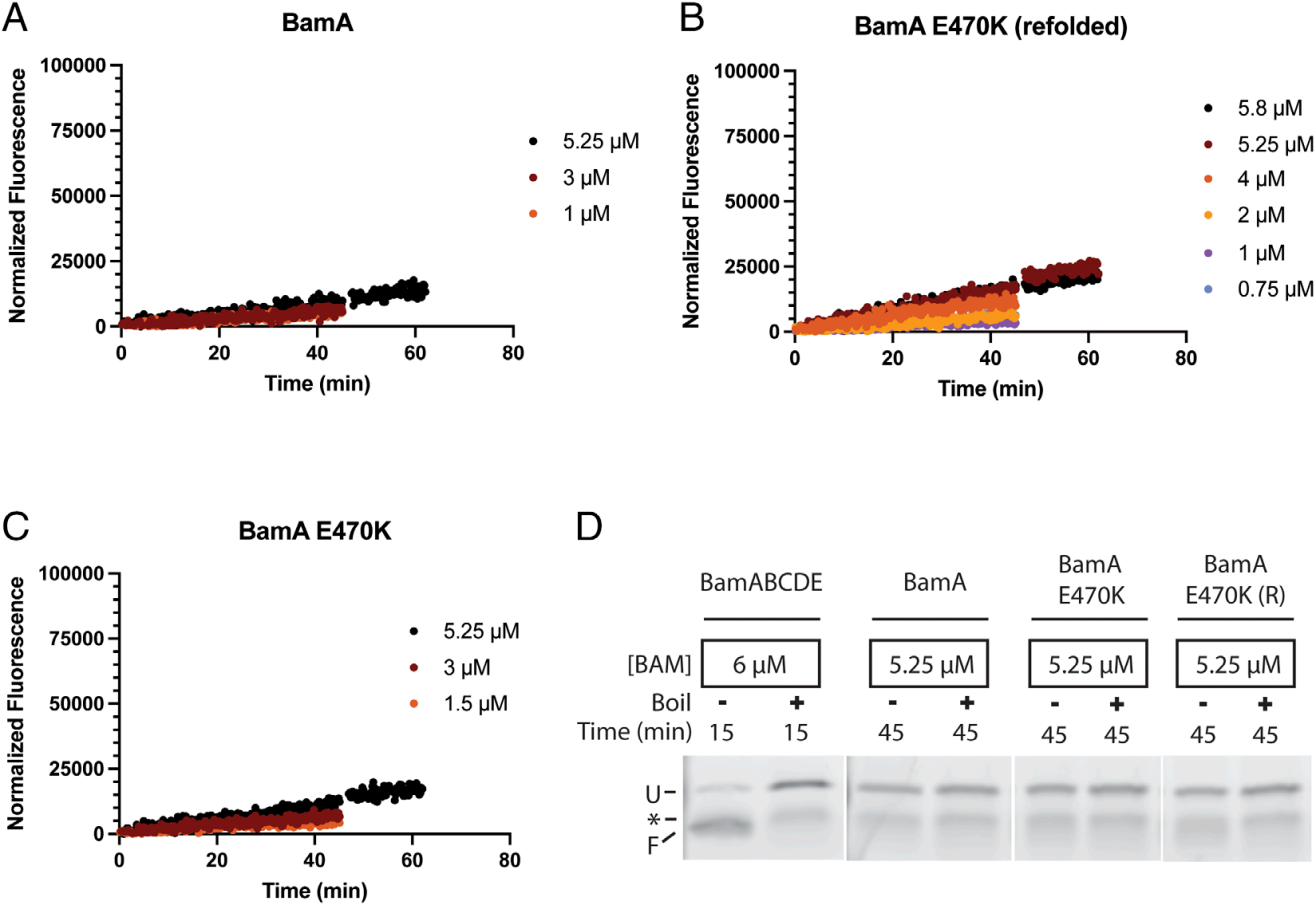
BamA alone is defective in activity. (*A*) Representative fluorescence vs. time data from a single-turnover activity assay of BamA alone. (*B* and *C*) Fluorescence vs. time data from single-turnover activity assays of refolded BamA(E470K) and membrane-extracted BamA(E470K), respectively. (*D*) Fluorescent images of SDS-PAGE of bOmpA-A488 folding reactions with BamABCDE complex, wild-type BamA alone, BamA(E470K) alone, and refolded BamA(E470K) (R). Indicated are unfolded (U) and folded (F) bOmpA-A488 and a fluorescent contaminant (asterisk).

We next tested the effect of removing all BAM lipoproteins. To probe the catalytic capability of BamA by itself, we expressed a His-tagged BamA that complements BamA depletion in *E. coli* indicating that the tag is well tolerated (34). Purification of this protein yielded BamA with only trace amounts of copurified endogenously expressed BamB, BamC, and BamD (*SI Appendix*, Fig. S3). Single-turnover kinetic assays with BamA in EPL liposomes show minimal changes in bOmpA-A488 fluorescence, indicative of poor-to-no folding (Fig. 3*A*), consistent across three independent biological replicates. SDS-PAGE analysis confirmed that the reconstituted BamA was folded (*SI Appendix*, Fig. S3), however bOmpA-A488 was not folded by BamA, while it is nearly completely folded under similar conditions with wild-type BamABCDE (Fig. 3*D*). These results indicate that at concentrations close to saturation for wild-type BamABCDE complex, BamA by itself is essentially incapable of catalyzing OMP folding in *E. coli* lipid membranes.

Silhavy et al. have recently reported a gain-of-function mutation in BamA (54). Cell-based assays suggest that BamA(E470K)BCDE complex is phenotypically indistinguishable from wild-type BamABCDE (55). However, cells expressing BamA(E470K) can survive depletion of the essential BamD in a BamBCE knockout strain, indicating that BamA(E470K) by itself is able to support cell growth (54, 56). Therefore, we hypothesized that BamA(E470K) may rescue the in vitro activity of BamA alone. To test this hypothesis, we first expressed and purified BamA(E470K) from inclusion bodies to limit copurification of trace amounts of lipoproteins, which may contribute background levels of activity. The purified protein was solubilized in tris-buffered urea, refolded in detergent, and subjected to size exclusion chromatography (*SI Appendix*, *SI Materials and Methods*). We also expressed BamA(E470K) with its signal sequence and purified it from *E. coli* membranes. Neither refolded nor membrane-extracted BamA(E470K) demonstrated heat modifiability, consistent with previous reports (*SI Appendix*, Fig. S4*A*) (55). However, the BamA(E470K) proteins eluted similarly to membrane-extracted wild-type BamA from size exclusion chromatography columns and circular dichroism demonstrated similar secondary structure as wild-type BamA (*SI Appendix*, Fig. S4*B*). Thus, our refolded and membrane-extracted samples of BamA(E470K) likely adopt the BamA native fold. Interestingly, single-turnover kinetics measurements of the refolded and membrane-extracted BamA(E470K) mutant alone showed little-to-no bOmpA-A488 folding activity (Fig. 3 *B* and *C*). Furthermore, a purified BamA(E470K)BCDE complex showed activity similar to wild-type BamABCDE complex in our single-turnover experiments (*SI Appendix*, Fig. S5), consistent with the similar phenotypes observed in cell-based assays (55). Together, these results indicate that the BamA(E470K) mutation does not dramatically enhance the BAM complex k_fold_ nor does it bring the K_0.5_ for BamA by itself to values similar to the wild-type BamABCDE complex.

### BamD and BamB Are Individually Sufficient to Rescue Activity of BamA.

**Fig. 4.**
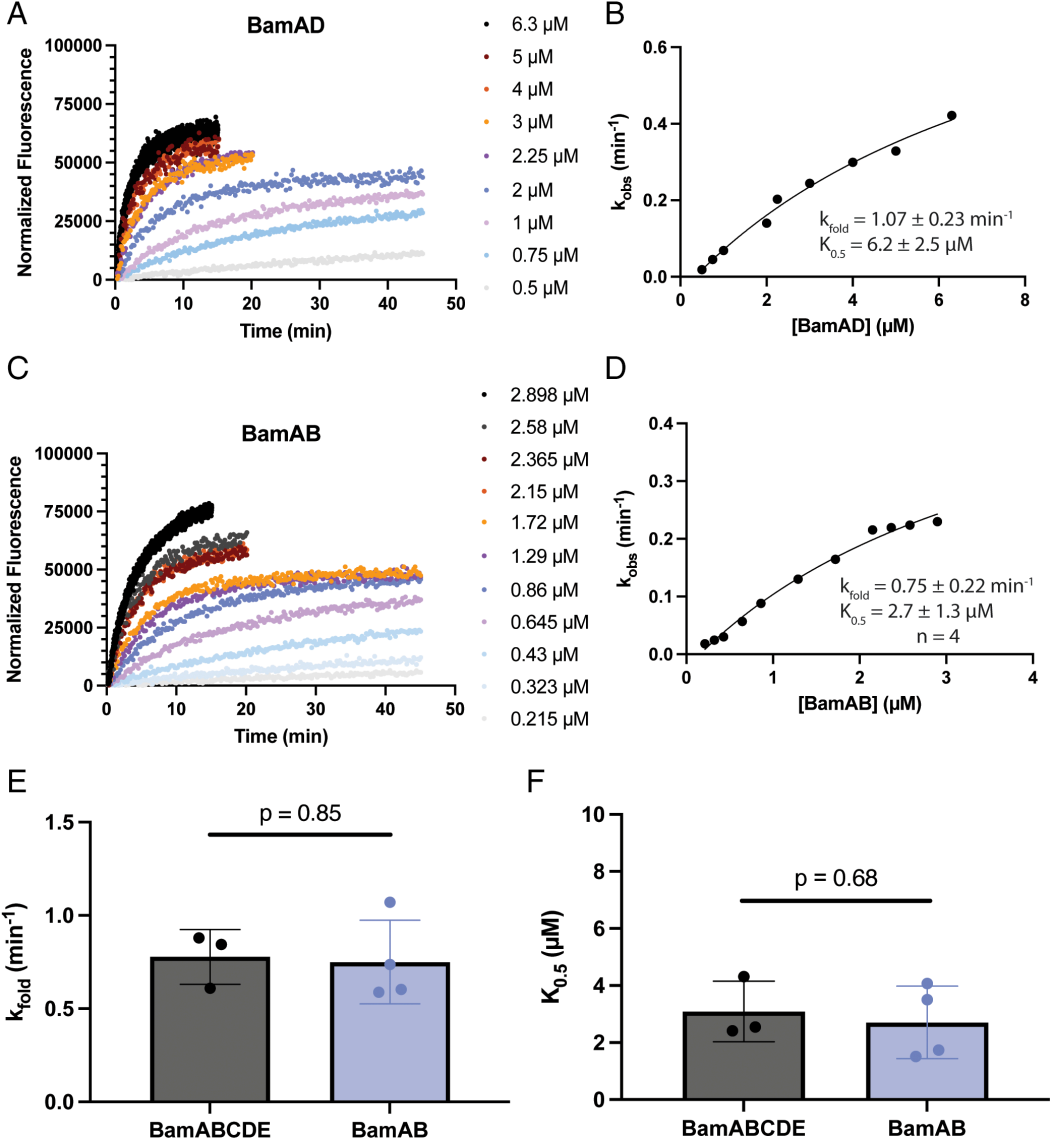
Both BamD and BamB can rescue activity of BamA alone. (*A*) Representative fluorescence vs. time data from a single-turnover activity assay of the BamAD subcomplex. (*B*) Data from (*A*) were fitted to Eq. **1**. *Inset*: values for k_fold_ and K_0.5_ represent the estimated value ± SE of the fit. Protein instability precluded extensive quantitative analysis. (*C*) Representative fluorescence vs. time data from a single-turnover activity assay of the BamAB subcomplex. (*D*) Data from (*C*) were fitted to Eq. **1**. *Inset*: fit parameters for four biological replicates, reported as the mean ± STD. (*E* and *F*) Calculated k_fold_ and K_0.5_ parameters, respectively, for WT BamABCDE and the BamAB subcomplex. An unpaired Welch’s *t* test indicated a *P*-value of 0.85 and 0.68, respectively, between WT BamABCDE (n = 3) and BamAB (n = 4). Concentrations used in the analysis for the BamAB subcomplex were normalized to the concentration of BamA in the sample (*SI Appendix*, *SI Materials and Methods*).

As BamA by itself proved to be inactive, we next sought to identify a minimal active BAM subcomplex. Because BamA and BamD are the only essential BAM components, we hypothesized that a BamAD subcomplex would be active enough to support cell growth (16, 17, 53). To test this, we coexpressed BamA with a His-tagged BamD. Purification resulted in a BamAD subcomplex (*SI Appendix*, Fig. S6) although multiple attempts produced low yields, which we attribute to poor expression and inherent protein instability, consistent with previous reports and the role of BamE in stabilizing BamAD interactions (18, 57). BamAD instability also resulted in poor liposome reconstitution yields despite multiple experimental attempts. Nevertheless, all BamAD reconstitutions displayed robust folding of bOmpA-A488 in the single-turnover assay, albeit only one reconstitution yielded enough material to sample the concentration range required to fit a square hyperbola (Fig. 4 *A* and *B*). Therefore, while the protein instability and poor proteoliposome reconstitution yields precluded rigorous quantification of kinetic parameters, BamAD demonstrated high levels of activity in all biological replicas qualitatively similar to wild-type BamABCDE. Furthermore, purification of BamADE subcomplexes resulted in more stable subcomplexes that also displayed levels of activity similar to wild-type BamABCDE complex (*SI Appendix*, Fig. S7). Thus, these results demonstrate that BamD can rescue activity of BamA alone in vitro.

To investigate whether BamD is necessary for activity, we coexpressed BamA with a His-tagged BamB. Purification resulted in a BamAB subcomplex (*SI Appendix*, Fig. S8). Remarkably, single-turnover kinetics assays with the BamAB subcomplexes reconstituted into EPL liposomes also displayed robust bOmpA-A488 folding activity (Fig. 4*C*). Fitting of the folding rate constants vs. BamAB concentration to a square hyperbola as above yielded a k_fold_ of 0.75 ± 0.22 min^−1^ and K_0.5_ of 2.7 ± 1.3 µM (mean ± STD from four independent BAM reconstitutions). Strikingly, the kinetic parameters are indistinguishable from those of wild-type BamABCDE (Fig. 4 *E* and *F*, *P* = 0.85 and *P* = 0.68, respectively) demonstrating that BamB is also able to rescue the activity of BamA alone.

### The BAM Complex Is Active In Vitro without the First Three POTRA Domains.

**Fig. 5.**
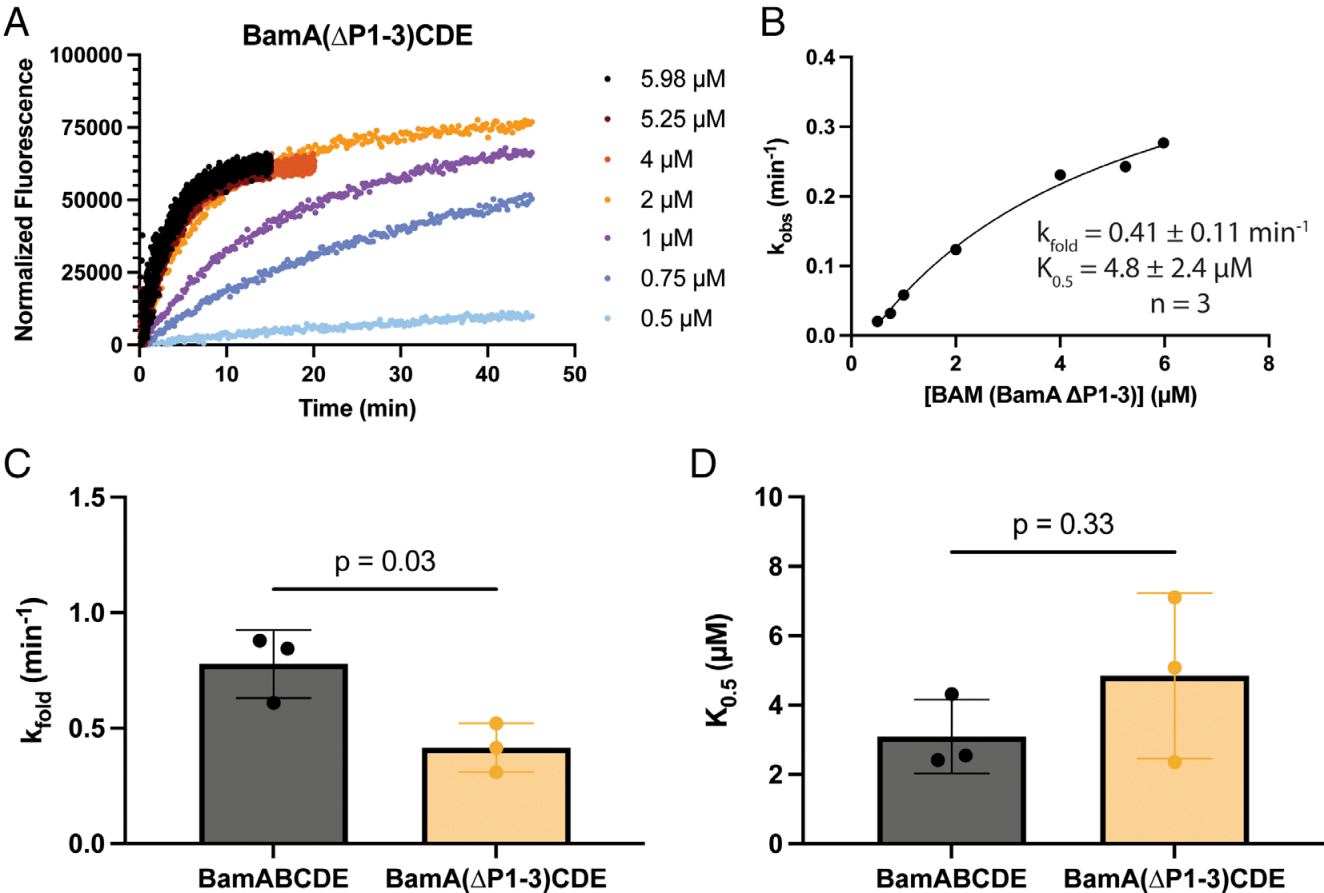
Single-turnover kinetics reveal that BAM is catalytically active without POTRA domains 1-3. (*A*) Representative fluorescence vs. time data from single-turnover activity assay of the BamA(∆P1-3)CDE mutant. (*B*) Data from (*A*) were fitted to Eq. **1**. *Inset*: fit parameters for three biological replicates, reported as the mean ± STD. (*C* and *D*) Calculated k_fold_ and K_0.5_ parameters, respectively for WT BamABCDE and BamA(∆P1-3)CDE. An unpaired Welch’s *t* test indicated a *P*-value of 0.03 and 0.33, respectively, between WT BamABCDE (n = 3) and BamA(∆P1-3)CDE (n = 3).

Having reported robust activity of wild-type BamABCDE, we next tested what role the POTRA domains play in OMP folding. We constructed a BAM mutant with a deletion of BamA POTRAs 1-3 (BamA ∆P1-3). Previous growth complementation assays show that deletion of POTRAs 1 and 2 are tolerated in *E. coli* but lead to growth defects, whereas deletion of POTRA3 is lethal (24, 58). Therefore, we hypothesized that deleting the first three POTRA domains would be deleterious to activity. SDS-PAGE confirmed purification of the expected BamA truncation, with minimal contamination from wild-type endogenous BamA (*SI Appendix*, Fig. S9). As expected, the ∆P1-3 mutant did not contain BamB, which interacts primarily with BamA POTRA3 (27), resulting in a BamA(∆P1-3)CDE complex. Single-turnover activity assays revealed that the BamA(∆P1-3)CDE complex was active, and substrate saturation was approached with the concentration of BAM tested (Fig. 5*A*). The rate constants were plotted as a function of BAM concentration and fitted to a square hyperbola as described above (Fig. 5*B*). Remarkably, the analysis revealed the ∆P1-3 mutant showed only a modest decrease in activity, with a k_fold_ of 0.41 ± 0.11 min^−1^ (mean ± STD from three independent BAM reconstitutions, Fig. 5*C*). Strikingly, the K_0.5_ of the BamA ∆P1-3 mutant was no different than wild-type BamABCDE (Fig. 5*D*), suggesting that removal of the three terminal POTRA domains, as well as BamB, has little effect on the substrate binding affinity. Similar results were obtained for BamA(∆P1)BCDE and BamA(∆P1-2)BCDE complexes (*SI Appendix*, Fig. S10 *A*–*D*), further illustrating the limited role of the POTRAs 1-3 in substrate binding and BAM catalysis under our experimental conditions.

## Discussion

The most frequently used approach to evaluate BAM complex folding of OMPs relies on their “heat modifiability” and the use of SDS-PAGE to separate folded and unfolded species (35). While accessible and in principle applicable to any OMP substrate, the approach is discontinuous, affords limited sampling of fast kinetics, and requires effective folding quenching and substantial postprocessing (SDS-PAGE and quantification). A commonly used alternative to measure BAM activity is based on folding OmpT, an OMP with protease activity which cleaves a quenched fluorescent peptide (36–41). The resulting increase in fluorescence over time can be monitored continuously with high sensitivity in this form of coupled assay. However, extracting the kinetic parameters for the BAM-catalyzed reaction is difficult as the first reaction (OmpT folding) produces the catalyst (OmpT) for the measured reaction (peptide cleavage), resulting in complex kinetic behavior. To address these challenges, we developed a fluorescent OMP folding reporter based on the β-barrel TM domain of OmpA labeled with Alexa Fluor 488 (bOmpA-A488). We demonstrate that bOmpA-A488 change in fluorescence robustly tracks its spontaneous folding into short-chain PC liposomes (evaluated by the standard SDS-PAGE assay) under multiple conditions where the intrinsic folding rates varied by over two orders of magnitude while showing no fluorescence change when no folding occurs (Fig. 1). Therefore, we attribute bOmpA-A488 change in fluorescence to an increase in its fluorescence quantum yield when the protein adopts its folded structure in the membrane.

A central challenge in measuring the folding activity of the BAM complex is the proclivity of its OMP substrates to aggregate or misfold, which compete with productive folding (59). This has hindered previous attempts to study BAM kinetics using steady-state approaches requiring saturating concentrations of substrate (45). To overcome this challenge, we took advantage of the high sensitivity of the bOmpA-A488 reporter to set up single-turnover reaction conditions with nanomolar concentrations of unfolded substrate and micromolar concentrations of BAM complex to minimize the aggregation problem. We observed a saturable increase in fluorescence that fit to single exponential rates and scaled hyperbolically with the concentration of BAM complex (Fig. 2). Fitting of these data to an offset hyperbolic equation (Eq. **1**) allows the estimation of the BAM complex catalytic folding constant k_fold_ of 0.78 ± 0.15 min^−1^ (mean ± STD from three independent BAM reconstitutions). This value may seem very slow compared with ~600 min^−1^ typical of enzymes that catalyze chemical reactions (60). However, we interpret the k_fold_ as the overall rate constant for the entire folding process catalyzed by the BAM complex as analysis of the reaction endpoints by SDS-PAGE indicated heat-modifiable, folded species of bOmpA-A488 (Fig. 2*A*). According to the current mechanistic model, OMP folding involves sequential threading of substrate β-hairpins in the BamA barrel gate to form hybrid barrels, followed by budding of the completed barrels into the membrane (61–64). This process is likely to be significantly slower than enzyme-catalyzed chemical conversions. In fact, our reported k_fold_ is approximately 10-fold faster than previous estimates (45) and is in excellent agreement with the BAM k_fold_ of ~0.6 min^−1^ estimated using deterministic and stochastic models to simulate rates of OMP folding needed to sustain normal doubling times in *E. coli* (53). Therefore, our reported k_fold_ is consistent with current estimates of what is required for normal physiological conditions.

In addition to the k_fold_, analysis of the single-turnover kinetic data also yields K_0.5_, which represents the combination of the k_fold_ and the substrate binding and unbinding constants (K_0.5_ = (k_-1_+k_fold_)/k_1_). Assuming that substrate unbinding is much faster than the folding (k_-1_>>k_fold_), a conservative assumption given the slow k_fold_ of 0.78 ±0.15 min^-1^, the K_0.5_ approximates the substrate K_d_. We observe a K_0.5_ of 3.1 ± 1.1 µM (mean ± STD from three independent BAM reconstitutions) for wild-type BamABCDE. As the BAM proteoliposomes reconstitution is the most challenging aspect to replicate from assay to assay, it likely introduces the most variability. Because we adapted our reconstitution methods from an established protocol (49), we expected similar orientation biases (the majority of BAM complex periplasmic components facing outward). Indeed, control accessibility assays revealed similar results for our reconstitutions (*SI Appendix*, Fig. S11). Nevertheless, different reconstitutions may result in variable yields of active enzyme, namely through differential complex orientation in the liposomes (i.e., POTRA domains facing outward vs. inward) or entrapment of liposomes inside other liposomes (i.e., multilamellar vesicles). While this variability is not expected to affect the k_fold_, an intrinsic property of the enzyme, different reconstitution efficiencies affect the estimate of K_0.5_, as this depends on the concentration of active enzyme. This is evidenced by a somewhat larger STD of the K_0.5_ estimates compared to the k_fold_ value. Nonetheless, the K_0.5_ of 3.1 ± 1.1 µM for BamABCDE is comparable to binding affinities of proteins in the OMP biogenesis pathway, where the reported K_d_ of SurA to tOmpA and SurA to BAM are 1.8 and 2.6 µM, respectively (12, 31). Taken together, this indicates that our observed kinetic parameters are physiologically reasonable. Thus, these experiments establish the use of the bOmpA-A488 folding reporter in combination with single-turnover kinetics as a robust platform to quantitatively test the activity of BAM.

To understand the effect of BamBCDE lipoproteins on the complex activity, we tested the activity of BamA by itself in liposomes composed of *E. coli* lipids. Several studies have previously demonstrated that BamA by itself can accelerate OMP folding into short-chain PC (12) and mixed PC/PE liposomes (45, 48, 65), suggesting BamA is responsible for driving the catalytic folding of OMPs. Notably, these liposomes with nonphysiological phospholipids allow OMP folding independent of BAM (45, 51, 65), which complicates extrapolation of folding rates to physiological conditions. Conversely, liposomes composed of *E. coli* polar lipids do not allow spontaneous insertion of OMPs (51). Therefore, we reconstituted BamA into liposomes composed of *E. coli* lipids such that any OMP folding activity could be ascribed to BamA. Unlike the wild-type BamABCDE complex, BamA by itself was unable to efficiently fold bOmpA-A488 in our single-turnover experiments (Fig. 3). This is consistent with previous reports of poor folding of tOmpA by BamA in EPL proteoliposomes (less than 20% after 2 h) (49) and is consistent with the lethality of BamD deletion in cells (17). However, BamA by itself has been reported to accelerate OMP folding into liposomes composed of synthetic short-chain (10 carbon) lipids with mostly phosphocholine (PC) head groups at pH 10 (48). We suggest that, under such conditions, BamA acceleration of OMP folding is mediated by local destabilization of the short-chain PC lipid membrane, rather than accommodation of substrate OMPs in the BamA lateral gate as is thought to happen under physiological conditions. This interpretation is consistent with results from our lab that showed BamA acceleration of OMP folding into short-chain PC lipids at pH 10 even when the lateral gate of BamA is locked by disulfides (65) As the gain-of-function mutant BamA(E470K) has been reported to bypass the essential requirement for BamD in *E. coli* (54, 56), we hypothesized that the BamA(E470K) mutant might be more active than the wild-type BamA in our assay. However, when reconstituted into *E. coli* phospholipid liposomes, BamA(E470K) by itself was just as defective as wild-type BamA at folding bOmpA-A488. Furthermore, BamA(E470K)BCDE complex did not show enhanced activity compared to wild-type BamABCDE complex. Combined, these results indicate that the E470K mutation does not dramatically improve the BAM complex k_fold_ nor does it appear to increase productive substrate binding as previously proposed (66).

To further probe the roles of the BAM lipoproteins, we sought to identify a minimal active BAM subcomplex. Besides BamA, BamD is the next most conserved protein of the BAM complex. It is essential for *E. coli* viability and has been proposed to play an essential role in OMP folding (16, 17), perhaps by binding substrate OMP (67, 68). It was thus not surprising that a BamAD subcomplex showed robust activity qualitatively similar to that of wild-type BamABCDE, albeit its intrinsic instability prevented rigorous quantification of its kinetic parameters (Fig. 4 *A* and *B*) (18, 57). As BamD coordinates binding of BamC and BamE to the complex, the only other possible BamA binary subcomplex is BamAB. Surprisingly, the single-turnover assay revealed that the BamAB subcomplex folding activity is the same as wild-type BamABCDE complex (Fig. 4 *E* and *F*). Contrary to the prevailing models, our results indicate that BamD is not essential for BAM-catalyzed OMP folding in vitro. Importantly, our findings are consistent with recent reports on the function of BamD. Specifically, Kumar and Konovalova demonstrated that the essential function of BamD is to prevent lethal jamming of the BamA pore by RcsF, rather than a direct involvement with OMP biogenesis (69). They also revealed that the BamA(E470K) mutation prevents RcsF jamming, thus bypassing the BamD deletion lethality in cells, which is also consistent with BamA(E470K) mutant not showing enhanced activity in our activity assays (Fig. 3 and *SI Appendix*, Fig. S5) Taken together, our results indicate that neither BamD nor BamB plays a significant role in OMP folding in our in vitro assay, and all the folding capacity is encoded in BamA. This is in accordance with a recent report by Noinaj et al. that in **Fusobacterium* nucleatum* OMP biogenesis is carried out by BamA without accessory lipoproteins (70). On the other hand, *E. coli* BamA alone was inactive in our in vitro assay. We suggest that this is due to isolated BamA reconstituted in liposomes adopting conformations that are incompatible with substrate binding and/or catalysis. Indeed, previous molecular dynamics simulations revealed that the POTRA domains of BamA alone interact extensively with the *E. coli* membrane surface, adopting conformations that are not observed when BamA is in complex with its accessory lipoproteins (71). Thus, we postulate that the presence of BamD or BamB stabilizes BamA conformations that allow OMP folding catalysis in vitro. In total, our findings challenge the current model that BamD plays an essential role in BAM activity and support alternative and somewhat overlapping roles for BamB and BamD as recently suggested (69).

Data from multiple labs suggested that BamA POTRA domains play a role in substrate binding (22, 24, 33). Specifically, because β-augmentation, or β-strand templating, was frequently observed for POTRA3 with neighboring POTRA domains in crystal lattices, it was hypothesized that one function of the POTRA domains might be to bind substrate OMPs and nucleate the formation of β-strands in a similar manner (22, 24). Furthermore, we previously identified that conformational flexibility between POTRAs 2 and 3 was important for *E. coli* cell growth (34). This led to the hypothesis that conformational ratcheting between POTRA domains may facilitate formation of β-hairpins between OMP β-strands bound to neighboring POTRA domains, thereby helping initial folding of OMPs. Moreover, the lethality of a POTRA3 deletion led to the hypothesis that POTRA3 may specifically serve an important role in OMP binding aside from its function to bind the nonessential lipoprotein BamB (24). To probe these hypotheses, we constructed BAM complexes with increasing severity of POTRA deletions: BamA(∆P1)BCDE, BamA(∆P1-2)BCDE, and BamA(∆P1-3)CDE. Based on the accepted model in the field that POTRA domains are important for binding OMPs, we predicted the POTRA deletions would have progressively bigger impacts on substrate binding. Additionally, because of the lethality in cells (24), the POTRA1-3 deletion, which also removes BamB from the BAM complex, was expected to have a severe impact on activity. However, single-turnover assays of the BamA(∆P1-3)CDE complex revealed a very modest impact on k_fold_ (Fig. 5*C*). We rationalize that the small reduction in k_fold_ is unlikely to be responsible for lethality because a 5-to-10-fold reduction in BamA expression is well tolerated in *E. coli* (72). Furthermore, control experiments with POTRA1 and POTRA1-2 deletion mutants also yielded k_fold_ values similar to those of the POTRA1-3 deletion mutant and those mutants are tolerated in cell growth assays (24, 58). The observed k_fold_ is also well within the range (between 0.18 and 3.6 min^−1^) predicted in OMP folding simulations to support normal cell growth (53). The data indicate that the first 3 POTRA domains do not play a substantial role in folding catalysis and does not support the POTRA ratcheting model of forming OMP β-hairpins. We also found no significant difference in K_0.5_, or apparent substrate binding affinity, between the complexes with POTRA1-3 deletions and wild-type BamABCDE (Fig. 5). Taken together, the data indicate that the first three POTRA domains do not significantly contribute to binding under our single-turnover conditions. While it is possible that the lethality of the POTRA3 deletion is due to a role in substrate turnover that is not captured in our assay, we propose a different alternative.

The prevailing model for OMP biogenesis calls for the chaperone SurA to capture nascent OMPs as they emerge from the Sec translocon in the inner membrane shuttling them to the BAM complex (32). However, SurA is not an essential protein in *E. coli* (32, 73) While other periplasmic chaperones such as Skp, DegP, and FkpA may be involved in aspects of OMP biogenesis, they do not seem to play a role in substrate delivery to the BAM complex. This suggests the existence of an alternative pathway for substrate delivery. There is growing evidence that the Sec translocon machinery directly interacts with the BAM complex to form a transperiplasmic bridge (58, 74–77). Using single-molecule tracking in live cells, we recently showed that diffusion of the Sec holotranslocon is unusually slow for an inner membrane protein, approaching that of the BAM complex in the outer membrane (58). However, Sec diffusion is significantly increased when the BAM complex has a POTRA1 or POTRA1-2 deletion suggesting a Sec–BAM interaction. Consistent with these experiments, we propose that the main role of POTRA1-3 domains is to support a connection between the BAM complex and the Sec holotranslocon allowing direct transfer of nascent OMPs to the outer membrane. Thus, we suggest a model where OMPs transfer to BAM via parallel pathways, one involving direct transfer through a Sec–BAM bridge (*SI Appendix*, Fig. S12), and another dependent on SurA to shuttle nascent OMPs that fail to engage the bridge back to BAM. In this model, disruption of one pathway leads to outer membrane defects and/or impaired growth, while disruption of both pathways is lethal. Such model is consistent with the observed mutant phenotypes. A SurA deletion is not lethal as it disrupts only one pathway. Similarly, POTRA1 or POTRA1-2 deletions are not lethal as they disrupt the bridge (58), but SurA chaperoning is still intact. Recent cryo-EM data indicate that SurA binding to BAM is mediated by interactions with BamB as well as β-augmentation of POTRA1 by the flexible, unstructured N terminus of SurA (30, 78). As β-augmentation interactions are sequence promiscuous, we speculated that in the POTRA1 and POTRA1-2 deletions, SurA may be able to dock to BAM via β-augmentation of POTRA2 or 3 with its flexible N terminus, while retaining interactions with BamB. However, if POTRA1-3 are deleted (which also removes BamB from the complex), the Sec–BAM bridge is disrupted, and SurA loses its BAM docking sites disrupting the chaperoning pathway resulting in a lethal phenotype.

A physiological role for a Sec–BAM transperiplasmic bridge in OMP biogenesis is supported by biochemical experiments in independent laboratories (74–77) as well as our live cell microscopy data (58). The in vitro single-turnover kinetics experiments presented here are consistent with a role for the first three POTRA domains of BamA in formation of such bridge rather than a role in substrate binding or folding catalysis. On the other hand, some estimates of the distance between the inner and outer membrane may be inconsistent with a direct interaction between Sec and BAM (79) (*SI Appendix*, Fig. S12). This may suggest that additional periplasmic components important for OMP biogenesis are yet to be found.

## Materials and Methods

Experimental procedures for cloning and protein expression and purification are provided in *SI Appendix*, *SI Materials and Methods*. Plasmids and oligonucleotides used are detailed in *SI Appendix*, Tables S1 and S2.

### Labeling of bOmpA 1 Cys N-Term with Alexa Fluor 488.

A guanidine-HCl stock of bOmpA 1 Cys N-term was diluted 10-fold into 8 M urea, 20 mM Tris, 1 mM EDTA, 0.05 mM [tris(2-carboxyethyl)phosphine] (TCEP), pH 7.3, to yield ~100 µM OMP. The protein was reduced with 30 mM TCEP at 21 °C for 30 min. Protein was acetone precipitated (protocol adapted from Thermo Scientific) to remove excess reducing agent. Pellet was washed with cold 1 mM EDTA pH 6 and redissolved in urea buffer. Alexa Fluor-488 C_5_ maleimide was added to protein at a molar ratio of about 5:1 dye to Cysteine and incubated for 1 h at 21 °C, followed by incubation with 2 mM N-ethylmaleimide (NEM) for 10 min to block unreacted cysteines. Two rounds of acetone precipitation were performed to remove unreacted dye. The pellet was washed with 100% ethanol, dried with nitrogen gas, and redissolved in 8 M urea, 25 mM glycylglycine pH 8. A NanoDrop was used to check protein and dye concentration with appropriate extinction coefficients. Labeled protein was aliquoted into small portions, snap frozen with liquid nitrogen, and placed at −70 °C until use.

### Folding of bOmpA 1 Cys N-Term-A488 into PC Liposomes.

Solutions of PC liposomes (prepared as described in *SI Appendix*, *SI Materials and Methods*), UltraPure BSA (AM2616, Invitrogen), urea buffer (8 M urea, 20 mM Tris pH 8), and 1× TBS (25 mM Tris pH 8, 150 mM NaCl) + 1 mM EDTA were made to desired concentration of liposomes. bOmpA 1 Cys N-term-A488 was added to liposomes solution with continuous mixing to initiate folding reactions. Final reaction concentrations were 0.8 mM PC lipids, 0.5 mg/mL BSA, 0.8 M urea, 50 nM OMP. Procedures for solution fluorescence measurements and quantification of folding of bOmpA-A488 by SDS-PAGE are detailed in *SI Appendix*, *SI Materials and Methods*.

### Single-Turnover BAM Activity Assays.

DDM solubilized BAM was reconstituted into liposomes using a dialysis method adapted from refs. 38 and 49. The resulting proteoliposomes were extruded, and final BAM concentration was assayed by BCA assay (a detailed description is provided in *SI Appendix*, *SI Materials and Methods*). Solutions of BAM proteoliposomes/SurA/1× TBS + 1 mM EDTA buffer were placed in cuvettes on the fluorimeter with stirring. Background fluorescence measurements were taken, as described in *SI Appendix*, *SI Materials and Methods*, then the reaction was initiated by the addition of 0.1 µM bOmpA-A488 in 8 M urea, 20 mM Tris pH 8, and fluorescence was monitored at appropriate times. Final reaction concentrations were 0.1 µM SurA, 0.01 µM bOmpA-A488, 0.8 M urea, and variable amounts of BAM proteoliposomes (the same proteoliposome reconstitution was used to conserve reconstitution efficiency across a single biological replicate). Processing of the data was performed as described in *SI Appendix*, *SI Materials and Methods* in GraphPad Prism 10. Activity curves were constructed by potting the single exponential folding rate constants (k_obs_) against the concentration of BAM. Activity is modeled after Scheme **1**, and activity curves were fitted to an adapted hyperbolic curve described by Eq. **1**:

[Scheme 1] $$uOMP+BAM\underset{k_{-1}}{\overset{k_{1}}{⇌}}uOMP-BAM\overset{k_{\mathrm{fold}}}{\to }fOMP+BAM,$$

[1] $$k_{\mathrm{obs}}=\frac{k_{\mathrm{fold}}*\left(\left[E\right]-F\right)}{K_{0.5}+\left[E\right]-F},$$

where [E] represents the BAM concentration, F represents the x-intercept “offset” (allows variation in BAM quantification by the BCA assay), and K_0.5_ is described by Eq. **2**:

[2] $$K_{0.5}=\frac{k_{-1}+k_{\mathrm{fold}}}{k_{1}}.$$

While all reported kinetic assays include 100 nM SurA (a 10-fold excess of OMP, which is standard for the field) (37, 46, 49), we did not expect it to have an effect given the low micromolar binding affinities of SurA to BAM and OMPs (12, 31). Control experiments without SurA indicated similar activity as with 100 nM SurA (*SI Appendix*, Fig. S13).

## Supplementary Material

Appendix 01 (PDF)

## Data Availability

The raw data associated with this study are publicly available in the CU Scholar repository at https://scholar.colorado.edu/concern/datasets/1544br23x (80). Study data are included in the article and/or *SI Appendix*.

## References

[1] T. J. Silhavy, D. Kahne, S. Walker, The bacterial cell envelope. Cold Spring Harb. Perspect. Biol. **2**, a000414 (2010). 20452953 10.1101/cshperspect.a000414 PMC2857177

[2] H. Nikaido, Molecular basis of bacterial outer membrane permeability revisited. Microbiol. Mol. Biol. Rev. **67**, 593–656 (2003). 14665678 10.1128/MMBR.67.4.593-656.2003 PMC309051

[3] R. Koebnik, K. P. Locher, P. Van Gelder, Structure and function of bacterial outer membrane proteins: Barrels in a nutshell. Mol. Microbiol. **37**, 239–253 (2000). 10931321 10.1046/j.1365-2958.2000.01983.x

[4] M. C. Sousa, New antibiotics target the outer membrane of bacteria. Nature **576**, 389–390 (2019). 31844257 10.1038/d41586-019-03730-x

[5] F.-U. Hartl, S. Lecker, E. Schiebel, J. P. Hendrick, W. Wickner, The binding cascade of SecB to SecA to SecYE mediates preprotein targeting to the E. coli plasma membrane. Cell **63**, 269–279 (1990). 2170023 10.1016/0092-8674(90)90160-g

[6] E. Schiebel, A. J. M. Driessen, F.-U. Hartl, W. Wickner, ΔμH+ and ATP function at different steps of the catalytic cycle of preprotein translocase. Cell **64**, 927–939 (1991). 1825804 10.1016/0092-8674(91)90317-r

[7] A. Tsirigotaki, J. De Geyter, N. Šoštaric, A. Economou, S. Karamanou, Protein export through the bacterial Sec pathway. Nat. Rev. Microbiol. **15**, 21–36 (2016). 27890920 10.1038/nrmicro.2016.161

[8] T. Krojer , Structural basis for the regulated protease and chaperone function of DegP. Nature **453**, 885–890 (2008). 18496527 10.1038/nature07004

[9] D. C. Marx , SurA is a cryptically grooved chaperone that expands unfolded outer membrane proteins. Proc. Natl. Acad. Sci. U.S.A. **117**, 28026–28035 (2020). 33093201 10.1073/pnas.2008175117 PMC7668074

[10] J. Qu, C. Mayer, S. Behrens, O. Holst, J. H. Kleinschmidt, The trimeric periplasmic chaperone Skp of Escherichia coli forms 1:1 complexes with outer membrane proteins via hydrophobic and electrostatic interactions. J. Mol. Biol. **374**, 91–105 (2007). 17928002 10.1016/j.jmb.2007.09.020

[11] U. Schäfer, K. Beck, M. Müller, Skp, a molecular chaperone of gram-negative bacteria, is required for the formation of soluble periplasmic intermediates of outer membrane proteins. J. Biol. Chem. **274**, 24567–24574 (1999). 10455120 10.1074/jbc.274.35.24567

[12] B. Schiffrin , Effects of periplasmic chaperones and membrane thickness on BamA-catalyzed outer-membrane protein folding. J. Mol. Biol. **429**, 3776–3792 (2017). 28919234 10.1016/j.jmb.2017.09.008 PMC5692476

[13] R. Wu, R. Stephenson, A. Gichaba, N. Noinaj, The big BAM theory: An open and closed case? Biochim. Biophys. Acta Biomembr. **1862**, 183062 (2020). 31520605 10.1016/j.bbamem.2019.183062 PMC7188740

[14] S. Wu , Interaction between bacterial outer membrane proteins and periplasmic quality control factors: A kinetic partitioning mechanism. Biochem. J. **438**, 505–511 (2011). 21671888 10.1042/BJ20110264

[15] A. M. Plummer, K. G. Fleming, From chaperones to the membrane with a bam! Trends Biochem. Sci. **41**, 872–882 (2016). 27450425 10.1016/j.tibs.2016.06.005 PMC5420074

[16] T. Wu , Identification of a multicomponent complex required for outer membrane biogenesis in Escherichia coli. Cell **121**, 235–245 (2005). 15851030 10.1016/j.cell.2005.02.015

[17] J. C. Malinverni , YfiO stabilizes the YaeT complex and is essential for outer membrane protein assembly in Escherichia coli. Mol. Microbiol. **61**, 151–164 (2006). 16824102 10.1111/j.1365-2958.2006.05211.x

[18] J. G. Sklar , Lipoprotein SmpA is a component of the YaeT complex that assembles outer membrane proteins in Escherichia coli. Proc. Natl. Acad. Sci. U.S.A. **104**, 6400–6405 (2007). 17404237 10.1073/pnas.0701579104 PMC1851043

[19] R. Voulhoux, M. P. Bos, J. Geurtsen, M. Mols, J. Tommassen, Role of a highly conserved bacterial protein in outer membrane protein assembly. Science **299**, 262–265 (2003). 12522254 10.1126/science.1078973

[20] N. Ruiz, B. Falcone, D. Kahne, T. J. Silhavy, Chemical conditionality: A genetic strategy to probe organelle assembly. Cell **121**, 307–317 (2005). 15851036 10.1016/j.cell.2005.02.014

[21] R. Albrecht , Structure of BamA, an essential factor in outer membrane protein biogenesis. Acta Crystallogr. Sect. D Biol. Crystallogr. **70**, 1779–1789 (2014). 24914988 10.1107/S1399004714007482

[22] P. Z. Gatzeva-Topalova, T. A. Walton, M. C. Sousa, Crystal structure of YaeT: Conformational flexibility and substrate recognition. Structure **16**, 1873–1881 (2008). 19081063 10.1016/j.str.2008.09.014 PMC2642521

[23] P. Z. Gatzeva-Topalova, L. R. Warner, A. Pardi, M. C. Sousa, Structure and flexibility of the complete periplasmic domain of BamA: The protein insertion machine of the outer membrane. Structure **18**, 1492–1501 (2010). 21070948 10.1016/j.str.2010.08.012 PMC2991101

[24] S. Kim , Structure and function of an essential component of the outer membrane protein assembly machine. Science **317**, 961–964 (2007). 17702946 10.1126/science.1143993

[25] N. Noinaj , Structural insight into the biogenesis of β-barrel membrane proteins. Nature **501**, 385–390 (2013). 23995689 10.1038/nature12521 PMC3779476

[26] Y. Gu , Structural basis of outer membrane protein insertion by the BAM complex. Nature **531**, 64–69 (2016). 26901871 10.1038/nature17199

[27] K. B. Jansen, S. L. Baker, M. C. Sousa, Crystal structure of BamB bound to a periplasmic domain fragment of BamA, the central component of the β-barrel assembly machine. J. Biol. Chem. **290**, 2126–2136 (2015). 25468906 10.1074/jbc.M114.584524 PMC4303665

[28] D. P. Ricci, C. L. Hagan, D. Kahne, T. J. Silhavy, Activation of the Escherichia coli β-barrel assembly machine (Bam) is required for essential components to interact properly with substrate. Proc. Natl. Acad. Sci. U.S.A. **109**, 3487–3491 (2012). 22331884 10.1073/pnas.1201362109 PMC3295296

[29] D. Bennion, E. S. Charlson, E. Coon, R. Misra, Dissection of β-barrel outer membrane protein assembly pathways through characterizing BamA POTRA 1 mutants of Escherichia coli. Mol. Microbiol. **77**, 1153–1171 (2010). 20598079 10.1111/j.1365-2958.2010.07280.x PMC2975826

[30] K. L. Fenn , Outer membrane protein assembly mediated by BAM-SurA complexes. Nat. Commun. **15**, 7612 (2024). 39218969 10.1038/s41467-024-51358-x PMC11366764

[31] B. Schiffrin , Dynamic interplay between the periplasmic chaperone SurA and the BAM complex in outer membrane protein folding. Commun. Biol. **5**, 560 (2022). 35676411 10.1038/s42003-022-03502-w PMC9177699

[32] J. G. Sklar, T. Wu, D. Kahne, T. J. Silhavy, Defining the roles of the periplasmic chaperones SurA, Skp, and DegP in Escherichia coli. Genes Dev. **21**, 2473–2484 (2007). 17908933 10.1101/gad.1581007 PMC1993877

[33] T. J. Knowles , Fold and function of polypeptide transport-associated domains responsible for delivering unfolded proteins to membranes. Mol. Microbiol. **68**, 1216–1227 (2008). 18430136 10.1111/j.1365-2958.2008.06225.x

[34] L. R. Warner, P. Z. Gatzeva-Topalova, P. A. Doerner, A. Pardi, M. C. Sousa, Flexibility in the periplasmic domain of BamA is important for function. Structure **25**, 94–106 (2017). 27989620 10.1016/j.str.2016.11.013 PMC5235167

[35] K. Nakamura, S. Mizushima, Effects of heating in dodecyl sulfate solution on the conformation and electrophoretic mobility of isolated major outer membrane proteins from Escherichia coli K-121. J. Biochem. **80**, 1411–1422 (1976). 828162 10.1093/oxfordjournals.jbchem.a131414

[36] C. L. Hagan, S. Kim, D. Kahne, Reconstitution of outer membrane protein assembly from purified components. Science **328**, 890–892 (2010). 20378773 10.1126/science.1188919 PMC2873164

[37] M. G. Iadanza , Lateral opening in the intact β-barrel assembly machinery captured by cryo-EM. Nat. Commun. **7**, 12865 (2016). 27686148 10.1038/ncomms12865 PMC5056442

[38] M. G. Iadanza , Distortion of the bilayer and dynamics of the BAM complex in lipid nanodiscs. Commun. Biol. **3**, 766 (2020). 33318620 10.1038/s42003-020-01419-w PMC7736308

[39] Y. Imai , A new antibiotic selectively kills Gram-negative pathogens. Nature **576**, 459–464 (2019). 31747680 10.1038/s41586-019-1791-1 PMC7188312

[40] M. Steenhuis , Combining cell envelope stress reporter assays in a screening approach to identify BAM complex inhibitors. ACS Infect. Dis. **7**, 2250–2263 (2021). 34125508 10.1021/acsinfecdis.0c00728 PMC8369490

[41] K. M. Storek , The Escherichia coli β-barrel assembly machinery is sensitized to perturbations under high membrane fluidity. J. Bacteriol. **201**, e00517-18 (2019). 30322857 10.1128/JB.00517-18 PMC6287456

[42] K. K. Andersen, H. Wang, D. E. Otzen, A kinetic analysis of the folding and unfolding of OmpA in urea and guanidinium chloride: Single and parallel pathways. Biochemistry **51**, 8371–8383 (2012). 22992178 10.1021/bi300974y

[43] E. J. Danoff, K. G. Fleming, Membrane defects accelerate outer membrane β-barrel protein folding. Biochemistry **54**, 97–99 (2015). 25513891 10.1021/bi501443p PMC4303321

[44] E. J. Danoff, K. G. Fleming, Novel kinetic intermediates populated along the folding pathway of the transmembrane β-barrel OmpA. Biochemistry **56**, 47–60 (2017). 28001375 10.1021/acs.biochem.6b00809 PMC5826654

[45] D. Gessmann , Outer membrane β-barrel protein folding is physically controlled by periplasmic lipid head groups and BamA. Proc. Natl. Acad. Sci. U.S.A. **111**, 5878–5883 (2014). 24715731 10.1073/pnas.1322473111 PMC4000854

[46] S. Hussain, H. D. Bernstein, The bam complex catalyzes efficient insertion of bacterial outer membrane proteins into membrane vesicles of variable lipid composition. J. Biol. Chem. **293**, 2959–2973 (2018). 29311257 10.1074/jbc.RA117.000349 PMC5827433

[47] G. J. Patel, S. Behrens-Kneip, O. Holst, J. H. Kleinschmidt, The periplasmic chaperone Skp facilitates targeting, insertion, and folding of OmpA into lipid membranes with a negative membrane surface potential. Biochemistry **48**, 10235–10245 (2009). 19780589 10.1021/bi901403c

[48] A. M. Plummer, K. G. Fleming, BamA alone accelerates outer membrane protein folding in vitro through a catalytic mechanism. Biochemistry **54**, 6009–6011 (2015). 26394056 10.1021/acs.biochem.5b00950 PMC4613867

[49] P. White , The role of membrane destabilisation and protein dynamics in BAM catalysed OMP folding. Nat. Commun. **12**, 4174 (2021). 34234105 10.1038/s41467-021-24432-x PMC8263589

[50] J. H. Kleinschmidt, Folding kinetics of the outer membrane proteins OmpA and FomA into phospholipid bilayers. Chem. Phys. Lipids **141**, 30–47 (2006). 16581049 10.1016/j.chemphyslip.2006.02.004

[51] N. K. Burgess, T. P. Dao, A. M. Stanley, K. G. Fleming, β-Barrel proteins that reside in the Escherichia coli outer membrane in vivo demonstrate varied folding behavior in vitro. J. Biol. Chem. **283**, 26748–26758 (2008). 18641391 10.1074/jbc.M802754200 PMC3258919

[52] A. H. Dewald, J. C. Hodges, L. Columbus, Physical determinants of β-barrel membrane protein folding in lipid vesicles. Biophys. J. **100**, 2131–2140 (2011). 21539780 10.1016/j.bpj.2011.03.025 PMC3149260

[53] S. M. Costello, A. M. Plummer, P. J. Fleming, K. G. Fleming, Dynamic periplasmic chaperone reservoir facilitates biogenesis of outer membrane proteins. Proc. Natl. Acad. Sci. U.S.A. **113**, E4794–E4800 (2016). 27482090 10.1073/pnas.1601002113 PMC4995976

[54] E. M. Hart, M. Gupta, M. Wühr, T. J. Silhavy, The gain-of-function allele bamA E470K bypasses the essential requirement for BamD in β-barrel outer membrane protein assembly. Proc. Natl. Acad. Sci. U.S.A. **117**, 18737–18743 (2020). 32675245 10.1073/pnas.2007696117 PMC7414184

[55] E. M. Hart , A small-molecule inhibitor of BamA impervious to efflux and the outer membrane permeability barrier. Proc. Natl. Acad. Sci. U.S.A. **116**, 21748–21757 (2019). 31591200 10.1073/pnas.1912345116 PMC6815139

[56] E. M. Hart, T. J. Silhavy, Functions of the BamBCDE lipoproteins revealed by bypass mutations in BamA. J. Bacteriol. **202**, e00401-20 (2020). 32817097 10.1128/JB.00401-20 PMC7549358

[57] S. Kumar, A. Konovalova, BamE directly interacts with BamA and BamD coordinating their functions. Mol. Microbiol. **120**, 397–407 (2023). 37455652 10.1111/mmi.15127 PMC10528117

[58] S. L. Upton, J. W. Tay, D. K. Schwartz, M. C. Sousa, Similarly slow diffusion of BAM and SecYEG complexes in live E. coli cells observed with 3d spt-PALM. Biophys. J. **122**, 4382–4394 (2023). 37853695 10.1016/j.bpj.2023.10.017 PMC10698321

[59] A. E. Tan, N. K. Burgess, D. S. DeAndrade, J. D. Marold, K. G. Fleming, Self-association of unfolded outer membrane proteins. Macromol. Biosci. **10**, 763–767 (2010). 20491126 10.1002/mabi.200900479 PMC3025446

[60] A. Bar-Even , The moderately efficient enzyme: Evolutionary and physicochemical trends shaping enzyme parameters. Biochemistry **50**, 4402–4410 (2011). 21506553 10.1021/bi2002289

[61] M. T. Doyle , Cryo-EM structures reveal multiple stages of bacterial outer membrane protein folding. Cell **185**, 1143–1156.e13 (2022). 35294859 10.1016/j.cell.2022.02.016 PMC8985213

[62] C. Shen , Structural basis of BAM-mediated outer membrane β-barrel protein assembly. Nature **617**, 185–193 (2023). 37100902 10.1038/s41586-023-05988-8

[63] D. Tomasek , Structure of a nascent membrane protein as it folds on the BAM complex. Nature **583**, 473–478 (2020). 32528179 10.1038/s41586-020-2370-1 PMC7367713

[64] R. Wu , Plasticity within the barrel domain of BamA mediates a hybrid-barrel mechanism by BAM. Nat. Commun. **12**, 7131 (2021). 34880256 10.1038/s41467-021-27449-4 PMC8655018

[65] P. A. Doerner, M. C. Sousa, Extreme dynamics in the BamA β-barrel seam. Biochemistry **56**, 3142–3149 (2017). 28569500 10.1021/acs.biochem.7b00281 PMC5995120

[66] T. M. A. dos Santos , Native β-barrel substrates pass through two shared intermediates during folding on the BAM complex. Proc. Natl. Acad. Sci. U.S.A. **121**, e2409672121 (2024). 39378083 10.1073/pnas.2409672121 PMC11494362

[67] C. L. Hagan, J. S. Wzorek, D. Kahne, Inhibition of the β-barrel assembly machine by a peptide that binds BamD. Proc. Natl. Acad. Sci. U.S.A. **112**, 2011–2016 (2015). 25646443 10.1073/pnas.1415955112 PMC4343090

[68] J. Lee , Substrate binding to BamD triggers a conformational change in BamA to control membrane insertion. Proc. Natl. Acad. Sci. U.S.A. **115**, 2359–2364 (2018). 29463713 10.1073/pnas.1711727115 PMC5877925

[69] S. Kumar, A. Konovalova, Bypassing BamD essentiality by mutations in a non-essential substrate. mBio **16**, e01769-25 (2025). 40823823 10.1128/mbio.01769-25 PMC12421809

[70] C. O. Cottom , Characterization of the OMP biogenesis machinery in Fusobacterium nucleatum. Structure **33**, 1878–1892.e5 (2025), 10.1016/j.str.2025.08.008. 40897170 PMC12646852

[71] P. J. Fleming , BamA POTRA domain interacts with a native lipid membrane surface. Biophys. J. **110**, 2698–2709 (2016). 27332128 10.1016/j.bpj.2016.05.010 PMC4919588

[72] S. K. Aoki , Contact-dependent growth inhibition requires the essential outer membrane protein BamA (YaeT) as the receptor and the inner membrane transport protein AcrB. Mol. Microbiol. **70**, 323–340 (2008). 18761695 10.1111/j.1365-2958.2008.06404.x PMC2579741

[73] P. E. Rouvière, C. A. Gross, SurA, a periplasmic protein with peptidyl-prolyl isomerase activity, participates in the assembly of outer membrane porins. Genes Dev. **10**, 3170–3182 (1996). 8985185 10.1101/gad.10.24.3170

[74] Y. Wang , A supercomplex spanning the inner and outer membranes mediates the biogenesis of β-barrel outer membrane proteins in bacteria. J. Biol. Chem. **291**, 16720–16729 (2016). 27298319 10.1074/jbc.M115.710715 PMC4974385

[75] S. Alvira , Inter-membrane association of the Sec and BAM translocons for bacterial outer-membrane biogenesis. eLife **9**, e60669 (2020). 33146611 10.7554/eLife.60669 PMC7695460

[76] M. L. Carlson , Profiling the Escherichia coli membrane protein interactome captured in Peptidisc libraries. eLife **8**, e46615 (2019). 31364989 10.7554/eLife.46615 PMC6697469

[77] D. Ranava , Lipoprotein DolP supports proper folding of BamA in the bacterial outer membrane promoting fitness upon envelope stress. eLife **10**, e67817 (2021). 33847565 10.7554/eLife.67817 PMC8081527

[78] P. A. Lehner , Architecture and conformational dynamics of the BAM-SurA holo insertase complex. Sci. Adv. **11**, eads6094 (2025). 40184469 10.1126/sciadv.ads6094 PMC11970506

[79] T. Lithgow, C. J. Stubenrauch, M. P. H. Stumpf, Surveying membrane landscapes: A new look at the bacterial cell surface. Nat. Rev. Microbiol. **21**, 502–518 (2023). 36828896 10.1038/s41579-023-00862-w

[80] W. N. Bergman, M. C. Sousa, Data from “Single-turnover kinetics of BAM activity with bOmpA-A488.” CU Scholar. https://scholar.colorado.edu/concern/datasets/1544br23x. Deposited 9 December 2025.

