# Supplementary material for: A fluorescent reporter and single-turnover kinetics reveal insight into BAM complex function
Source: Proc Natl Acad Sci U S A. 2025 Dec 24;122(52):e2514687122. doi: 10.1073/pnas.2514687122 (PMC12772190; doi:10.1073/pnas.2514687122)
Supplement: Supplementary file 1 — Appendix 01 (PDF) [file pnas.2514687122.sapp.pdf]

PNAS

Supporting Information for
A fluorescent reporter and single-turnover kinetics reveal new insight into BAM complex function.

Whitney Nicole Bergman[a], and Marcelo Carlos Sousa[a,1]
[a]Department of Biochemistry, University of Colorado Boulder, Boulder, Colorado 80309

[1]To whom correspondence should be addressed:
Marcelo C. Sousa
596 UCB
3415 Colorado Avenue
Boulder, CO 80309
Email: Marcelo.Sousa@colorado.edu

This PDF file includes:

SI Materials and Methods
Figures S1 to S13
Tables S1 to S2
SI References

Supporting Information Materials and Methods

Cloning of bOmpA 1 Cys N-term

An N-terminal cysteine was introduced between residues 2-3 in bOmpA (residues 22-197 of WT full length OmpA plus an N-terminal methionine). Plasmids and primers are provided in Tables S1 and S2. This resulted in a construct of mature bOmpA 1 Cys N-term without a signal sequence for expression into inclusion bodies.

Expression of Unfolded OMP Inclusion Bodies

For expression into inclusion bodies, *E. coli* BL21 (DE3) cells were transformed with plasmid containing bOmpA 1 Cys N-term gene and plated onto LB agar plates supplemented with 50 µg/mL kanamycin. Colonies were used to inoculate 5 mL of LB supplemented with kanamycin and grown overnight at 37°C. 1.25 mL of the overnight starter culture was used to inoculate 100 mL of fresh LB supplemented with kanamycin and cells were grown at 37°C until the culture reached an OD_{600} of 0.4-0.6. Protein expression was induced with 1 mM isopropyl β-D-thiogalactopyranoside (IPTG) and incubated at 37°C for 3 hours. Cells were harvested by centrifugation and pellets were resuspended in 30 mL 50 mM Tris pH 8, 0.5 mg/mL lysozyme. Cell solution was frozen at -70°C until use.

Purification of Unfolded OMP from Inclusion Bodies

To purify unfolded OMP, frozen cell solutions were thawed in a warm water bath and benzonase nuclease was added. Solutions were sonicated at 70% power for pulses of 5 seconds with 30 second breaks for a total pulse time of 2 minutes. Insoluble material was pelleted by centrifugation at 7650g for 30 minutes at 4°C and pellets were resuspended in 30 mL 50 mM Tris pH 8, 0.5% Triton X-100. Resuspensions were sonicated at 40% power for pulses of 10 seconds with 20 second breaks for a total pulse time of 2 minutes. Insoluble material was pelleted by another round of centrifugation. Pellets were resuspended in 30 mL 10 mM Tris pH 8, 1 mM ethylenediaminetetraacetic acid (EDTA) and pelleting and resuspension was repeated 3 times to remove residual detergent. On the last resuspension, pellet was resuspended in 1.25 mL EDTA buffer supplemented with 1 mM phenylmethylsulfonyl fluoride (PMSF), insoluble material was pelleted again, supernatant was discarded, and pellets were placed at -20°C until use. To prepare solubilized denatured OMP, the purified inclusion body pellet was resuspended in 1.5 mL 6 M guanidine-HCl, 25 mM Tris pH 8, 2 mM TCEP, and solution was filtered with a 0.1 µm filter. Protein solution was snap-frozen in liquid nitrogen and stored at -70°C until use.

Preparation of Lipid Films

Phosphatidylcholine (PC) 10:0 (850325, Avanti Research), PC 12:0 (850335, Avanti Research), PC 18:1 (Δ9-cis) (DOPC, 850375, Avanti Research), and *E. coli* Extract Polar (100600, Avanti Research) lipids were purchased as 25 mg/mL stocks in chloroform. For pure lipid films, an appropriate volume of lipid stock was aliquoted into glass vials to obtain 10 mM or 8 mg/mL lipids for PC or *E. coli* lipids, respectively, when resuspended in a volume of 250 µL. Lipids were dried under a flow of nitrogen gas to form a thin lipid film, and residual chloroform was evaporated off by vacuum desiccation overnight. Dried lipid films were stored at -20°C under nitrogen gas. For PC 10:0/PC 12:0 mixtures, appropriate volumes of pure chloroform lipid stocks were added to form 3:1, 1:1, or 1:3 molar ratios of PC 10:0 to PC 12:0 such that the total lipid concentration when resuspended in 250 µL is 10 mM. Films were formed and dried as above.

Preparation of PC Liposomes

PC liposomes were made by rehydrating lipid films in 250 µL 1X Tris-Buffered Saline (TBS) + EDTA (25 mM Tris pH 8, 150 mM NaCl, 1 mM EDTA) at 21°C for 30 minutes to make 10 mM lipid solutions. Solutions were bath sonicated for 15 minutes, allowed to rest for 10 minutes, vortexed at power 6 for 10 seconds, then extruded through a 0.2 µm filter at least 21 times using a mini-extruder (Avanti Research).

Fluorescence Measurements of tOmpA-A488

All solution fluorescence measurements were performed with the PTI (Photon Technology International) steady-state spectrofluorometer QuantaMaster (Model QM-6) (Horiba) controlled with the PTI FeliX32 Version 1.2 (2001-2005) software. The instrument was set to a PMT of 1000, excitation wavelength of 485 nm, emission wavelength of 516 nm, 3 nm monochromator slit widths, and a circulating water bath set to 21°C. Real time corrections were enabled and source gain was referenced to 485 nm and set to 1 V. Folding assays were prepared, as described above, and placed in 10 mm rectangular quartz cuvettes (3-Q-10, Starna Cells, Inc) with stir bars. Background measurements were taken for all folding reaction solutions before OMP was added. Folding reactions were initiated by addition of bOmpA-A488 in urea and fluorescence measurements were taken at appropriate times. Processing of the data was performed as described below (Post-Processing of bOmpA-A488 Fluorescence Measurements) and data were plotted as the change in fluorescence against time. Curves were fitted to double or single exponential functions with Prism 10 software.

Quantification of Folding of bOmpA-A488 by SDS-PAGE
To analyze extent of folding of bOmpA-A488, samples from folding reactions described above were quenched with 4X SDS-PAGE loading buffer (200 mM Tris-HCl, 8% SDS, 6 mM bromophenol blue, 4.3 M glycerol, 0.57 mM 2-mercaptoethanol) and kept on ice until ready to run on gel. For time courses, two samples were taken at the last timepoint, and one was boiled at 95°C for 5 min to denature the protein. All samples were loaded onto 12% (w/v) Tris-HCl SDS-PAGE gels and ran at 200V on ice. Gels were imaged using an Amersham Typhoon 5 (Cytiva) in fluorescence mode under a 488 nm laser with a Cy2 filter, a pixel size of 50 or 100 μm, and the PMT set to 465 V. Progression of folding was quantified as an increase in the folded bOmpA-A488 band intensity with the ImageJ software (Fiji). Briefly, equal-sized boxes were drawn around the entire lane for each sample in the gel. Within the Analyze->Gels->Plot Lanes function, a baseline was drawn for the total lane fluorescence, and vertical lines were drawn to delimit the folded band peak. Only the folded band intensity was considered to eliminate complications from previously observed higher molecular weight aggregates, which would contribute to a loss in the unfolded band intensity (1). Folding was quantified by normalizing the fluorescence of the folded band peak to the total lane fluorescence and data were plotted as the change in normalized folded band intensity from the first time point against time.

Post-Processing of bOmpA-A488 Fluorescence Measurements
For PC liposome reactions: all time points were adjusted for the delay in reading the first timepoint after the reaction was initiated, 6 points for each time point (collected as intervals of 2 points/second over 3 seconds) from measurements were averaged (except for the continuously read, PC 10:0 condition), and background fluorescence values were subtracted from each measurement. The initial timepoint fluorescence was subtracted from each timepoint to obtain a change in fluorescence.
For BAM proteoliposome single-turnover reactions: all time points were adjusted for the delay in reading the first timepoint after the reaction was initiated, and background fluorescence values were subtracted from each measurement. Reaction curves were fitted to single exponential functions in Prism 10 and outliers were detected and eliminated from the fit using the default parameters (Q = 1%). The predicted y-intercept was used to normalize the values to get an estimate of the fluorescence change from time = 0.

Expression and Purification of SurA
For expression of SurA, *E. coli* Rosetta (DE3) cells were transformed with a plasmid coding for the SurA protein with an N-terminal 6-histidine tag (pMS 332) and plated onto LB agar supplemented with 50 μg/mL kanamycin. A colony was used to inoculate a 50 mL LB + kanamycin (50 μg/mL) culture and cells were grown overnight at 37°C with shaking. 1L cultures of LB + kanamycin (50 μg/mL) were inoculated with the overnight culture and allowed to grow at 37°C until reaching an OD_{600} of 0.6. 1 mM IPTG was added to cultures to induce expression, and cells were incubated for 5 hours at 37°C. Cells were harvested by centrifugation at 4,000g for 20 minutes. Cell pellets were resuspended in lysis buffer (25 mM Tris pH 7.5, 300 mM NaCl) supplemented with a protease

inhibitor cocktail (EDTA acid-free, Roche) and stored at -80°C overnight. Cells were thawed and lysed by sonication with 10 second pulses for a total pulse time of 2 minutes. Lysis was spun at 16,000g for 30 minutes to pellet cell debris. The supernatant was loaded onto a Ni-NTA column equilibrated with lysis buffer. The column was washed with one column volume of lysis buffer, then 10 column volumes of wash buffer (25 mM Tris pH 7.5, 300 mM NaCl, 20 mM imidazole). The protein was eluted from the column with elution buffer (25 mM Tris pH 7.5, 300 mM NaCl, 250 mM imidazole), and fractions containing the protein were pooled.

Cloning of BAM Complex Mutants

The parent plasmid containing all the BAM complex genes (BamABCDE-8His) pJH114 was kindly provided by Harris Bernstein and used for subsequent cloning. Plasmids and primers/gene fragments used are provided in Tables S1 and S2. For POTRA deletion mutants, gene fragments were ordered from Twist Bioscience with sequences overlapping BamA, excluding codons for V26 through R91 (for ΔP1), V26 through E171 (for ΔP1-2), or V26 through I260 (for ΔP1-3). Gibson assembly was used to combine these gene fragments with the WT BamABCDE plasmid backbone (created by digestion with restriction enzymes KasI and AleI) For construction of BamA-His plasmid, a gene fragment was ordered from Twist Bioscience covering all the BamA gene of the WT BamABCDE plasmid, with a 6xHis2xAla tag inserted at amino acid position 22 and a XbaI restriction site after the BamA gene. This fragment was digested with restriction enzymes KasI and XbaI and ligated into the similarly digested WT BamABCDE plasmid backbone. For the BamA(E470K)BCDE mutant, site-directed mutagenesis was performed using primers upper 2804 and lower 2804 against a pET vector containing WT BamA (pMS 1224). The resulting clone sequence was confirmed by sequencing, then digested with restriction enzymes BstBI and NcoI and ligated into the similarly digested WT BamABCDE plasmid backbone. For the BamA-His(E470K) mutant, the plasmids for the BamA-His and the BamA(E470K)BCDE mutants were digested with restriction enzymes BstBI and NcoI, and the digested BamA(E470K) insert was ligated into the BamA-His digested backbone. For the BamADE mutant, the BamD and BamE genes were amplified using primers upper 2805 and lower 2805 against a BamABDE plasmid template (pJH114 with a deletion of the BamC gene). The resulting PCR product was digested with restriction enzymes KflI and XbaI, then ligated into a similarly digested BamABDE plasmid backbone. For the BamAD mutant, a gene fragment was ordered from Twist Bioscience covering the BamD gene, with a C-terminal AALE linker + a 8xHis tag and flanked by KflI and XbaI restriction sites. This gene fragment was digested with restriction enzymes KflI and XbaI, then ligated into a similarly digested BamABDE plasmid backbone. For the BamAB mutant, a gene fragment was ordered from Twist Bioseceince covering the BamA and BamB genes, with a XbaI restriction site after the BamB gene. This gene fragment was digested with restriction enzymes BstBI and XbaI and ligated into the similarly digested WT BamABCDE plasmid backbone. All ligation and Gibson cloning products were cloned into *E. coli* XL-10 Gold competent cells and clones were confirmed by whole plasmid sequencing (Plasmidsaurus Inc).

Expression and Purification of BAM Complex Mutants

For expression of BAM complex mutants, BL21 (DE3) cells were transformed with the appropriate plasmid and plated onto LB agar plates supplemented with 100 µg/mL ampicillin. Colonies were used to inoculate 6 mL of LB supplemented with ampicillin and incubated for 7 hours at 37°C. Cells were pelleted, media decanted, and cells were resuspended in fresh LB media. 1 mL of washed cells were used to inoculate 1L of LB supplemented with 100 µg/mL ampicillin and lactose autoinduction supplement (0.6% glycerol, 0.05% glucose monohydrate, 0.2% lactose monohydrate) and cells were grown overnight with shaking at 37°C. Cells were harvested by centrifugation, pellets were resuspended in 10 mL lysis buffer (50 mM Tris pH 8, 2 mM EDTA, 0.5 mg/mL lysozyme, protease inhibitor cocktail) per 1L culture, and cell solutions were frozen at -70°C. Cell solutions were thawed in a warm water bath and supplemented with Benzonase nuclease, then lysed with an Emulsiflex C3 Homogenizer (Avestin). Solutions were centrifuged at 7650g for 30 min at 4°C to pellet cell debris, then the supernatant was spun at 256,630g for 2 hours at 4°C. The resulting membrane pellet was resuspended in 10 mL extraction buffer (25 mM Tris pH 8, 150 mM NaCl, 1% n-dodecyl-β-D-maltoside (DDM)) per gram of wet membrane and incubated with

rocking overnight at 4°C. Extraction was centrifuged again at 256,630g for 1.5 hours at 4°C to pellet insoluble content and the supernatant was filtered to 0.22 μm then affinity purified with a 5 mL HisTrap FF on the AKTA Pure FPLC system. Briefly, the extraction was loaded onto the column equilibrated with Buffer A (25 mM Tris pH 8, 150 mM NaCl, 0.5 mM TCEP, 0.03% DDM) +5% Buffer B (Buffer A + 500 mM imidazole), the column was washed with 4 column volumes 5% Buffer B, then the protein was eluted over a gradient of 5-100% Buffer B over 5 column volumes. Fractions containing protein were pooled, concentrated to ~10 mL (Vivaspin 20 100K MWCO PES concentrator), filtered to 0.22 μm, then gel filtered with a HiLoad 26/600 Superdex 200 pg column in 25 mM Tris pH 8, 150 mM NaCl, 0.5 mM TCEP, 0.03% DDM. Fractions containing protein were pooled and concentrated with a Vivaspin concentrator. *Optional for BamAB subcomplexes*: between the affinity purification and size exclusion steps, fractions containing protein were pooled, concentrated, then diluted 5-fold with 25 mM Tris pH 8, 0.5 mM TCEP, 0.03% DDM. Sample was filtered to 0.22 μm, then purified with ion exchange chromatography with a 1 mL MonoQ 5/50 GL on the AKTA Pure FPLC system. Briefly, the sample was loaded onto the column equilibrated with 25 mM Tris pH 8, 0.5 mM TCEP, 0.03% DDM, the column was washed with 5 column volumes buffer, then the protein was eluted in stepwise gradients with 25 mM Tris pH 8, 0.5 mM TCEP, 1M NaCl, and 0.03% DDM. Fractions were analyzed by SDS-PAGE to exclude samples with free BamB. Fractions containing enriched BamAB subcomplexes were pooled and subjected to size exclusion chromatography, as above.

Protein concentrations were measured using a NanoDrop with A_{280} measurements. Extinction coefficients were calculated with ProtParam (Expasy) as 291650 $M^{-1}cm^{-1}$ for WT BAM, BAM (BamA ΔP1), and BAM (BamA E470K), 287180 $M^{-1}cm^{-1}$ for BAM (BamA ΔP1-2), 249250 $M^{-1}cm^{-1}$ for BAM (ΔBamC), 205820 $M^{-1}cm^{-1}$ for BAM (BamA ΔP1-3), 140040 $M^{-1}cm^{-1}$ for BamA-His, BamA-His E470K, and refolded BamA E470K, 186340 $M^{-1}cm^{-1}$ for BamADE-His, 174880 $M^{-1}cm^{-1}$ for BamAD-His, and 202950 $M^{-1}cm^{-1}$ for BamAB-His. Protein quality was checked by running purified samples on 4-20% Mini-PROTEAN TGX Protein Gels (Bio-Rad) and staining with Coomassie. Protein stocks were then used immediately or snap frozen with liquid nitrogen and stored at -70°C until use.

Expression, Purification, and Refolding of Unfolded BamA E470K from Inclusion Bodies

BamA E470K was expressed into inclusion bodies and purified as described above. To refold BamA E470K, the inclusion body pellet was dissolved in 8M urea, 20 mM Tris pH 8, 1 mM TCEP, filtered with a 0.1 μm filter, and 2 mL of the BamA E470K stock in urea was added slowly to 18 mL 25 mM Tris pH 8, 150 mM NaCl, 1 mM TCEP, 1% DDM with constant stirring at room temperature for 24 hours. To purify refolded BamA E470K, the solution was spun at 20,000g in a tabletop centrifuge to pellet aggregates. The supernatant was filtered to 0.1 μm, then subjected to size-exclusion chromatography and processed as described above for BAM complex mutants.

Circular Dichroism of BamA Samples

To obtain CD spectra of the BamA samples, BamA, BamA E470K, and refolded BamA E470K were diluted into 10 mM phosphate pH 8, 0.03% DDM to final concentrations of 0.05 mg/mL protein in 5 mM NaCl. A blank solution and BamA samples were read on the Applied Photophysics Chirascan Plus CD and Fluorescence Spectrometer in a 1 mm quartz cuvette, measured from 185-200 nm. The pathlength and protein concentration were used to convert measurements to mean residue molar ellipticity (MRME).

Reconstitution of BAM Complex into E. coli Polar Liposomes

Briefly, *E. coli* polar lipid (EPL) films were rehydrated with 1X TBS + EDTA buffer (25 mM Tris pH 8, 150 mM NaCl, 1 mM EDTA) supplemented with 0.1% DDM to a concentration of 6 mg/mL EPL, vortexed to assist rehydration, allowed to incubate at room temperature for at least 10 minutes, vortexed again, then extruded through a 0.2 μm filter. EPL solutions were mixed with BAM solutions (maintaining a ratio around 10 μM BAM to 3.33 mg/mL EPL) and dialyzed against 1X TBS + EDTA buffer at room temperature for 2 days with a total of 4 buffer exchanges using Slide-A-Lyzer MINI 20K MWCO, 2 mL Dialysis Devices with continuous magnetic stirring in the buffer chamber. Proteoliposomes were extruded first through a 1 μm filter about 5 times, then through a 0.2 μm filter about 7 times to homogenize liposome solution and remove lipid aggregates. This step is crucial

to avoid unwanted fluorescence signal in folding assays due to OMP aggregation at lipid aggregates. Final concentration of BAM proteoliposomes was quantified using a Rapid Gold BCA Protein Assay Kit (Pierce) with BSA as the standard, according to the supplier protocol. Molecular weights used to convert to molar concentrations were 199892.73 Da for WT Bam ABCDE, 199891.79 Da for BamA(E470K)BCDE, 192627.5 Da for BamA(ΔP1)BCDE, 183837.49 Da for BamA(ΔP1-2)BCDE, 133775.39 Da for BamA(ΔP1-3)CDE, 89320.05 Da for BamA-His, 89319.11 Da for BamA-His E470K, 88485.29 Da for folded BamA E470K, 125775.81 Da for BamADE-His, 115750.62 for BamAD-His, and 129813.38 for BamAB-His. Empty EPL liposomes were prepared as above, except instead of adding BAM, 1X TBS + EDTA supplemented with DDM was added to give a final added concentration of DDM of 0.167%, which amounts to 327:1 mole DDM:mole BAM (this is a ballpark ratio for detergent occupancy on membrane proteins (2), and is supported by measurements of the total DDM in a BAM purification, see below).

Quantification of DDM in Wildtype BamABCDE

A protocol for quantification of DDM was adapted from (3). Briefly, the reaction is initiated by mixing 72 µL sample with 8 µL 20% 2,6 dimethylphenol in 100% ethanol and 240 µL concentrated sulfuric acid in SafeSeal Eppendorf tubes. The mixture is inverted to mix, then incubated at room temperature for 40 minutes to allow the reaction to cool. Solutions were then transferred to a 96-well plate (Corning Assay Plate with Low Evaporation Lid, Cat. 3370), and absorbance was read at 510 nm. Standard curves were created with buffer containing 25 mM Tris pH 8, 0.5 mM TCEP, and 0-0.014% DDM. 10-40-fold dilutions of wildtype BamABCDE complex were assayed to determine the DDM concentration in the stocks.

SDS-PAGE Heat Modifiability Assays to Check Foldedness of BamA and bOmpA-A488

BAM complex stock solutions and samples from single-turnover activity assays were routinely subject to SDS-PAGE to test for "heat modifiability" of BamA and bOmpA-A488. For this, sample was added to 4X SDS-PAGE loading buffer and kept on ice or boiled at 95°C for 5 minutes. Samples were either loaded onto 12% (w/v) Tris-HCl SDS-PAGE gels (for detecting bOmpA-A488 folding) or 4-20% Mini-PROTEAN TGX Protein Gels (Bio-Rad), then ran at 200V on ice. Gels were imaged using an Amersham Typhoon 5 (for bOmpA-A488 folding) or coomassie stained.

Protease Accessibility of Reconstituted BAM Complex

5 µM wildtype BamABCDE complex reconstituted in EPL liposomes was digested with 0.5 µg Pierce Trypsin Protease, MS grade at a protein:Trypsin ratio of 200:1 after 15 minutes of a single-turnover folding assay. 5 µM wildtype BamABCDE complex stock in DDM, used as a solubilized control, was also digested with 0.5 µg Trypsin. Digestion reactions were incubated at 37°C for 1.5 hour, then reactions were quenched by boiling at 95°C in SDS-PAGE loading buffer. Digestion reactions and 5 µM undigested BamABCDE complex stock in DDM were loaded onto 12% (w/v) Tris-HCl SDS-PAGE to analyze extent of digestion.

Estimation of BamAB Subcomplex Concentration by SDS-PAGE Densitometry

To estimate the concentration of BamAB subcomplexes (in samples with excess BamB), 3 µM BamAB stock solutions and 3 µM wildtype BamABCDE stock solutions were analyzed by SDS-PAGE using heat modifiability protocols. BamA in each sample was quantified with the ImageJ software as described above. For a given sample, the intensities of the folded and unfolded BamA bands were averaged for the cold and boiled samples, respectively. The relative BamA intensities between stock solutions were used to normalize the BamAB concentration to the amount of BamA present in the wildtype BamABCDE stock. These corrections were applied to normalize BamAB concentration for activity kinetics curves, accounting for the lower BamAB subcomplex concentrations tested.

Figures

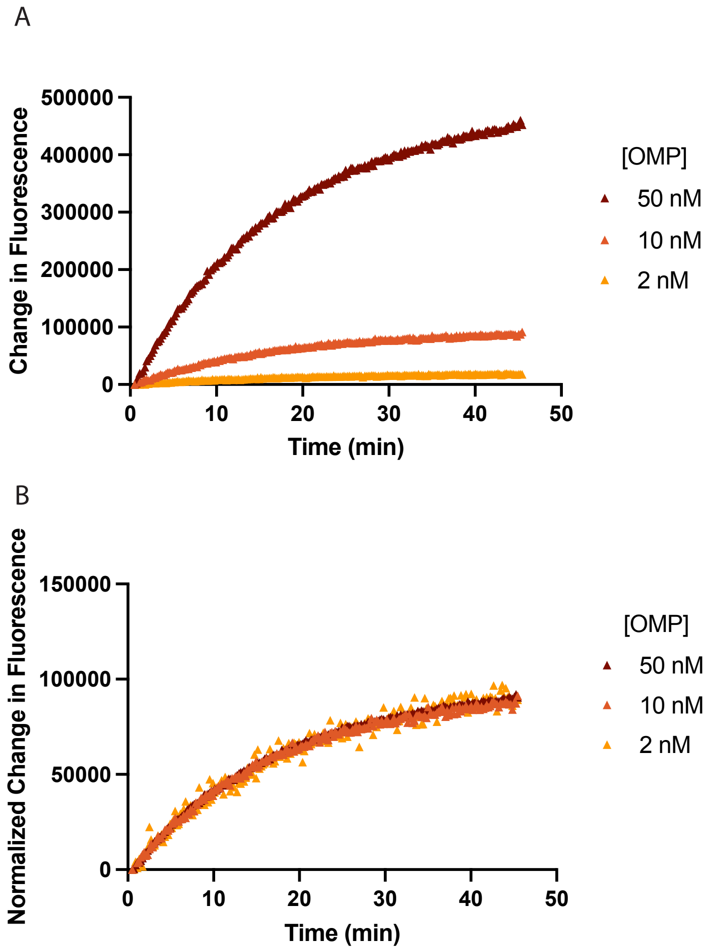

Fig. S1. Effect of changing the bOmpA-A488 concentration under single-turnover activity assay conditions. (A) Fluorescence of bOmpA-A488 folding with 0.75 µM BamABCDE proteoliposomes. (B) Fluorescence from (A) corrected by concentration of bOmpA-A488 normalized to the 10 nM OMP condition.

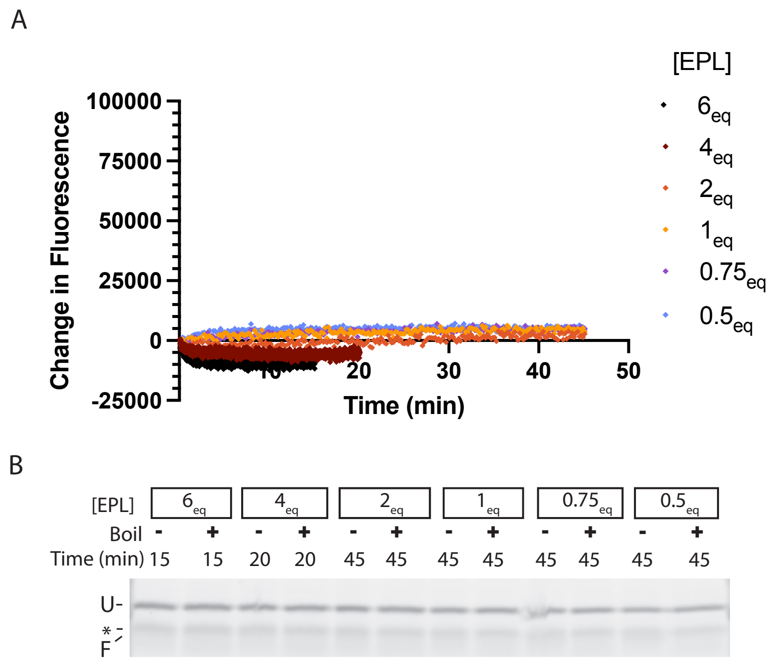

Fig. S2. bOmpA-A488 shows no folding in reconstituted empty EPL liposomes. (A) Fluorescence of bOmpA-A488 in solutions of empty EPL liposomes at roughly equivalent (eq) micromolar concentrations of BAM proteoliposomes typically used. (B) Fluorescent image of an SDS-PAGE heat modifiability assay of reactions from (A). Indicated are unfolded (U) and folded (F) bOmpA-A488, and a non-specific fluorescent contaminant (asterisk).

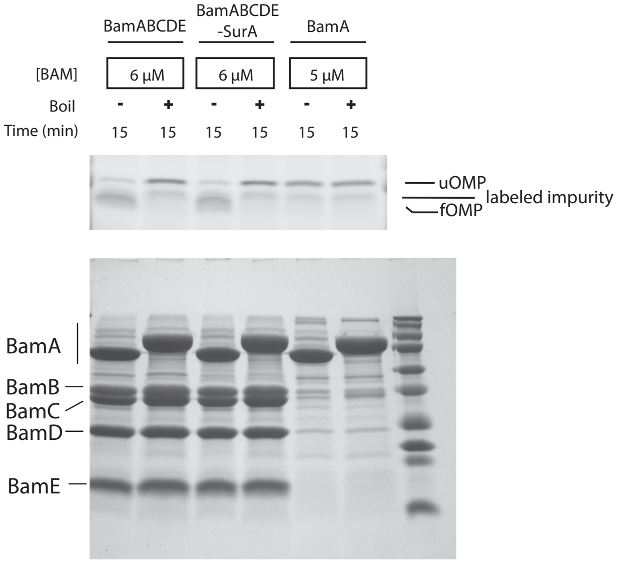

Fig. S3. SDS-PAGE confirms that reconstituted BamA is folded but not active. (Top) Fluorescent image of an SDS-PAGE heat modifiability assay of reactions with BamABCDE or BamA only after 15 minutes. Indicated are unfolded (uOMP) and folded (fOMP) bOmpA-A488, and a non-specific fluorescent impurity. (Bottom) Coomassie-stained gel from (A). BamA only is folded and has trace amounts of lipoproteins.

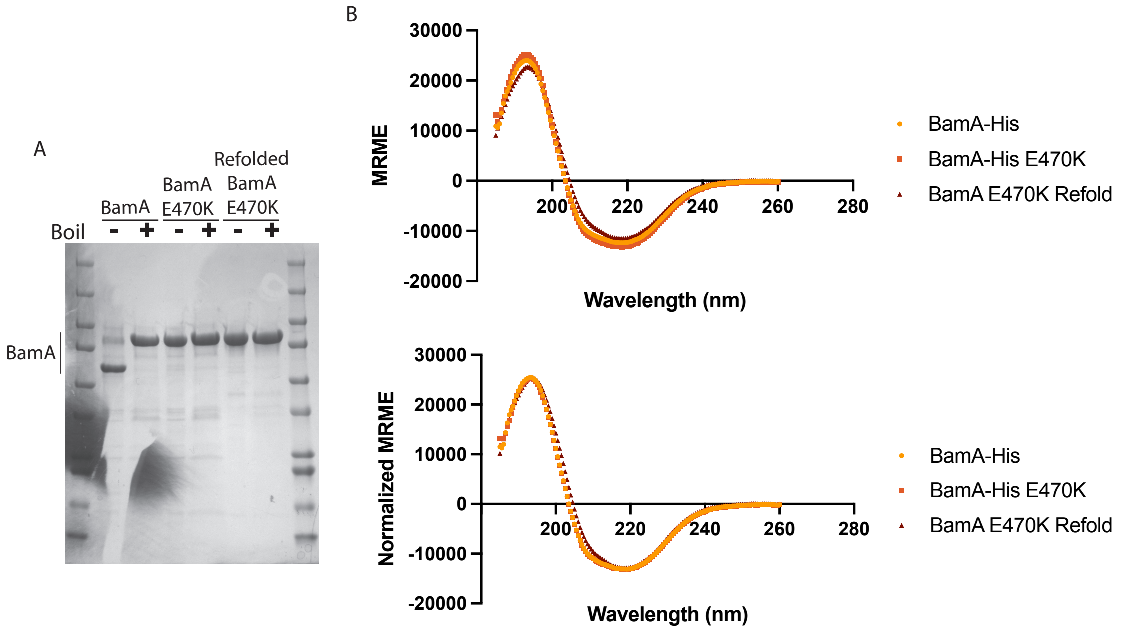

Fig. S4. Refolded BamA(E470K) adopts a similar structure to membrane-extracted BamA. (A) SDS-PAGE of purified BamA, BamA(E470K), and refolded BamA(E470K). (B) Circular dichroism spectra of protein samples from (A) reported as the mean residue molar ellipticity (MRME) or the Normalized MRME. Curves were normalized to BamA(E470K) by a multiplication factor of 1.06 and 1.12 for BamA and refolded BamA(E470K), respectively.

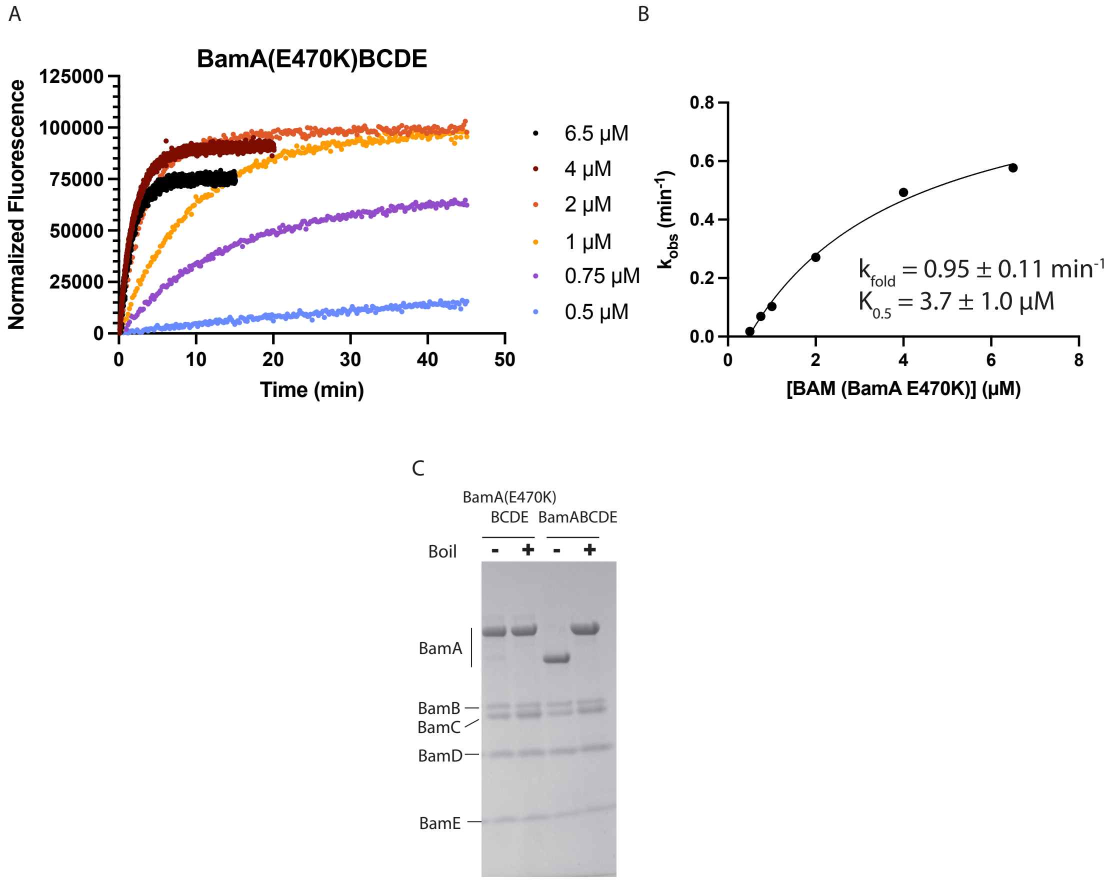

Fig. S5. Activity assay of BamA(E470K)BCDE shows activity comparable to WT BamABCDE. (A) Fluorescence data from one independent single-turnover activity assay of the BamA(E470K)BCDE mutant at different concentrations. (B) Single exponential rate constants from (A) were plotted against BAM concentration and fitted to Eq. 1 and represents one biological replicate. Inset values for k_{fold} and $K_{0.5}$ represent the estimated value ± SE of the fit. (C) SDS-PAGE of purified BamA(E470K)BCDE compared to wildtype BamABCDE.

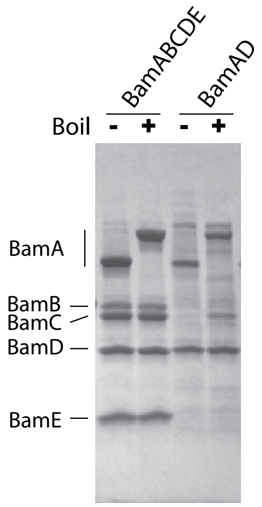

Fig. S6. Purification of BamAD mutant subcomplex. SDS-PAGE of purified BamABCDE complex and BamAD subcomplex. BamA displays “heat modifiability” in both complexes.

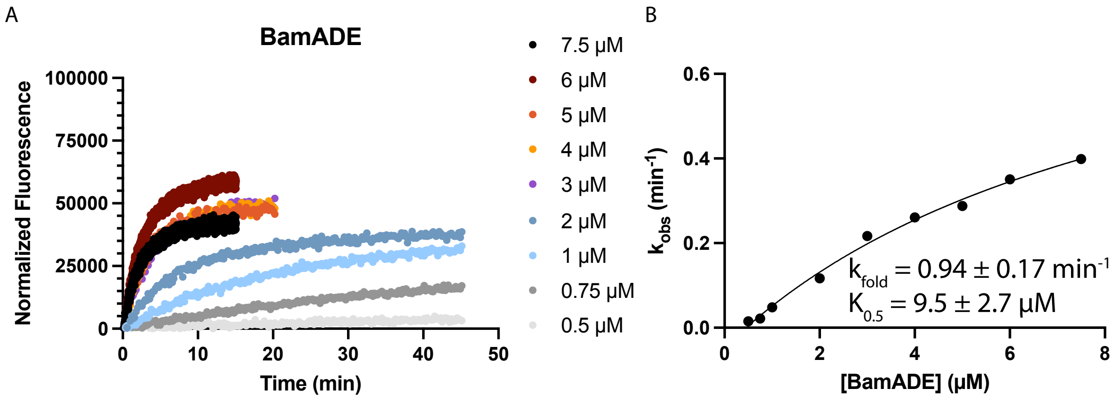

Fig. S7. Activity assay of BamADE shows activity comparable to WT BamABCDE. (A) Fluorescence data from one independent single-turnover activity assay of the BamADE subcomplex at different concentrations. (B) Single exponential rate constants from (A) were plotted against BAM concentration and fitted to Eq. 1 and represents one biological replicate. Inset values for k_{fold} and $K_{0.5}$ represent the estimated value ± SE of the fit.

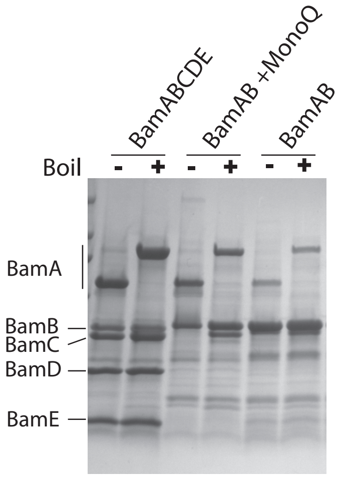

Fig. S8. Purification of BamAB subcomplexes produce excess free BamB lipoprotein. SDS-PAGE of purified BamAB subcomplex shows excess BamB is present (lanes 5-6) compared to the typical BamA:BamB stoichiometry in WT BamABCDE complex (lanes 1-2). Addition of an ion exchange (MonoQ) purification step to the BamAB purification protocol removes excess BamB (lanes 3-4).

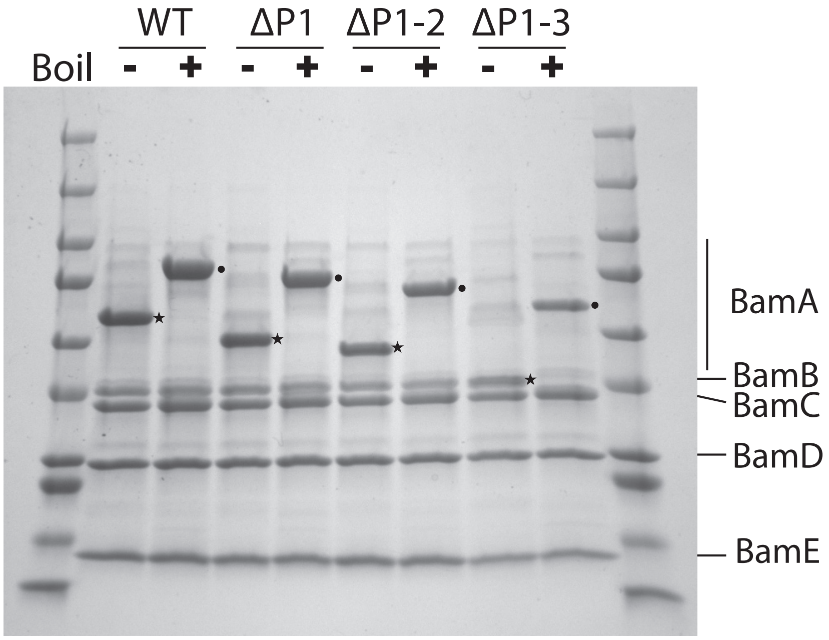

Fig. S9. Purification of BAM POTRA deletion mutants. SDS-PAGE of purified BAM complexes with BamA truncations. Highlighted are the change in molecular weight of the BamA proteins, while BamB, BamC, BamD, and BamE are also present. Note that for the ∆P1-3 mutant, folded BamA runs at a similar molecular weight as BamB. Folded and unfolded BamA bands are indicated with stars and circles, respectively.

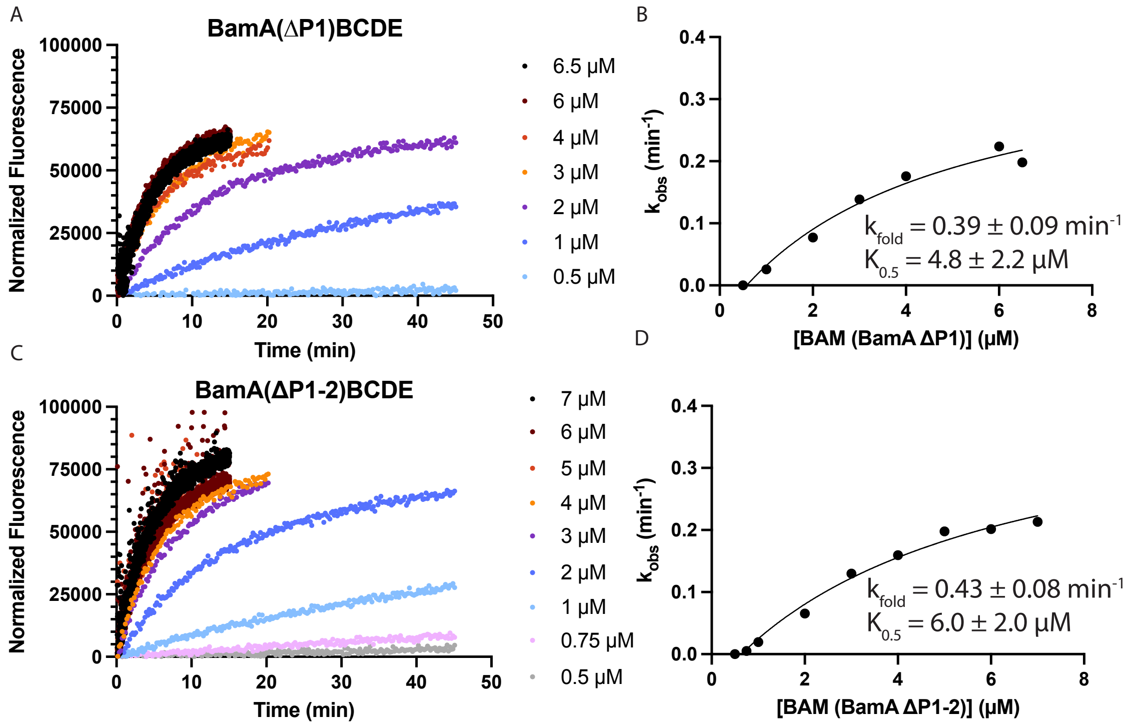

Fig. S10. Single-turnover activity assays of BamA(ΔP1)BCDE and BamA(ΔP1-2)BCDE. (A) Fluorescence data from one independent single-turnover activity assay of the BamA(ΔP1)BCDE mutant at different concentrations. (B) Single exponential rate constants from (A) were plotted against BAM concentration and fitted to Eq. 1 and represents one biological replicate. Values for k_{fold} and $K_{0.5}$ represent the estimated value ± SE of the fit. Fluorescence data and fitted rate constants are shown in (C-D) for the BamA(ΔP1-2)BCDE mutant.

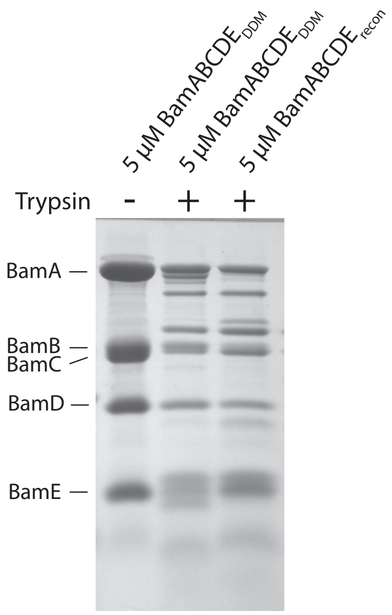

Fig. S11. Limited protease digestion reveals outward-facing BamABCDE reconstituted in EPL liposomes. SDS-PAGE of trypsin treated BamABCDE reconstituted in EPL liposomes indicates that the periplasmic side is facing outwards. Note, similar levels of BamA and BamBCDE lipoproteins of reconstituted BamABCDE (right lane) as BamABCDE in detergent (DDM) (middle lane), compared to input protein levels (left lane). Samples were incubated with 0.5 μg trypsin at 37°C for 1.5 hours prior to SDS-PAGE analysis.

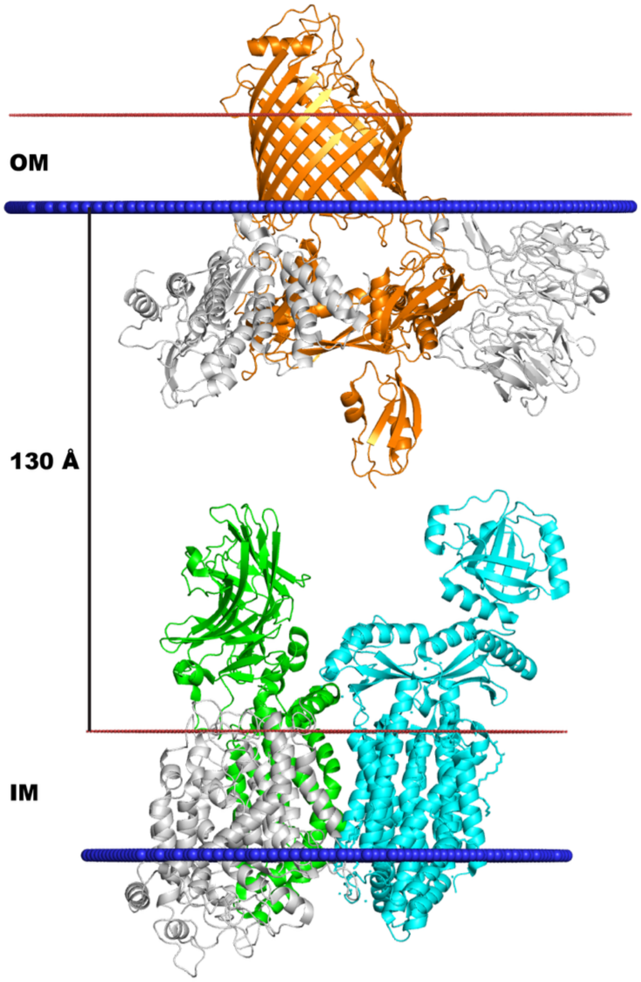

Fig. S12. Models of the SecYEGDF-YidC holotranslocon and the BAM complex in the same scale with the inner and outer membrane separated by 130 Å. A composite model of the holotranslocon was contructed from the cryo-EM model (PDB_ID: 5MG3) embedded in an inner membrane (IM) representation as estimated by the PPM server (4). The models of YidC (PDB_ID: 6AL2) (5) and SecDF in its "I" conformation (PDB_ID: 5XAP) (6) were superimposed in the transmembrane helices of the corresponding proteins in the cryoEM holotranslocon model to yield the composite model shown. SecYEG is colored gray, YidC is green, and SecDF is cyan. The periplasmic domain of SecDF protrudes approximately 60 Å from the inner membrane. This is consistent with atomic force microscopy (AFM) studies that measured a protrusion of ~63 Å assigned to the "I" conformation of SecDF (7). The model of the BAM complex is from a cryo-EM structure in a frequently observed conformation (PDB_ID: 7BNQ) embedded in an outer membrane (OM) representation as estimated by the PPM server (8). BamA is shown in orange while the accessory lipoproteins BamBCDE are shown in gray. The periplasmic domains of BamA protrude approximately 70 Å from the inner leaflet of the outer membrane. In the figure, the IM and OM are separated by 130 Å in the same scale as the protein models. There are several published estimates of the periplasmic thickness. Early ultrastructure imaging of *E. coli* cells indicated a thickness of 130 Å (9) while another study reported a range of 106 to 146 Å (10). Other estimates are derived from structures of protein complexes that span the periplasm. These include values of 125 to 145 Å from Wza-Wzc (11) and ~150 Å from the Type Three secretion system (12). A study imaging hydrated cross sections of bacteria report a periplasmic thickness estimate of 209.9 ± 26.9 Å for "uncompressed sections" although other sections were thinner (13).

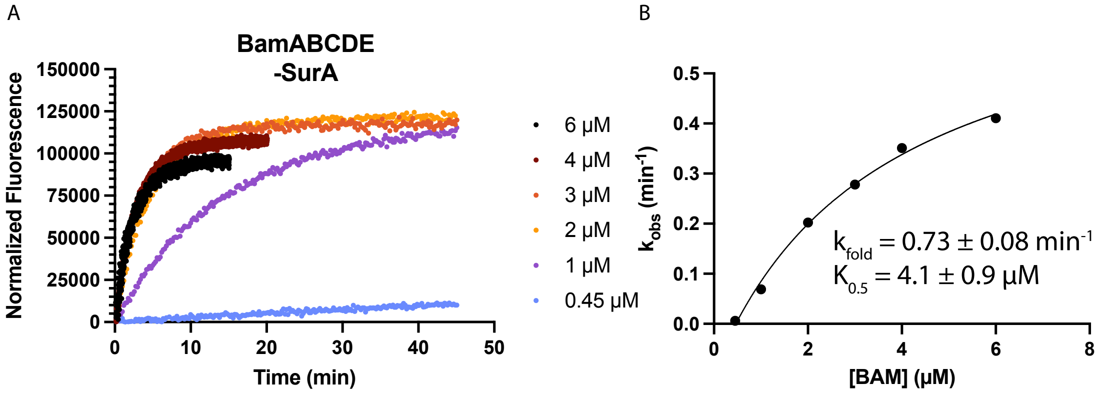

Fig. S13. Single-turnover activity of BamABCDE without SurA is similar to BamABCDE with 0.1 μM SurA. (A) Fluorescence data from one independent single-turnover activity assay of BamABCDE at different concentrations, without the addition of SurA. (B) Single exponential rate constants from (A) were plotted against BAM concentration and fitted to Eq. 1 and represents one biological replicate. Inset values for k_{fold} and $K_{0.5}$ represent the estimated value ± SE of the fit.

Tables

Table S1. Plasmids used in this study.

Plasmid	Internal Code	Description	Reference
bOmpA	pMS 471	pET28a (+) vector containing residues 22-197 of *E. coli* OmpA with an added N-terminal Met	This study
bOmpA 1 Cys N-term	pMS 755	bOmpA plasmid pMS 471 with a cysteine added between the residues corresponding to residues 22-23 of *E. coli* OmpA	This study
SurA	pMS 332	pET vector containing WT *E. coli* SurA gene	This study
WT BamABCDE	pMS 1289	pJH114, plasmid with genes encoding all five subunits of the *E. coli* BAM complex	(14)
BamA(ΔP1)BCDE	pMS 1843	WT BamABCDE plasmid with BamA gene missing codons V26-R91	This study
BamA(ΔP1-2)BCDE	pMS 1844	WT BamABCDE plasmid with BamA gene missing codons V26-E171	This study
BamA(ΔP1-3)BCDE	pMS 1845	WT BamABCDE plasmid with BamA gene missing codons V26-I260	This study
BamA-His	pMS 1846	WT BamABCDE plasmid with an N-terminally 6-His tagged BamA gene (coding sequence identical to BamA in complementation plasmid (15)), and deletion of BamBCDE lipoproteins	This study
BamA	pMS 1224	Plasmid with gene encoding mature *E. coli* BamA (codons E22-W810) for expression into inclusion bodies	(16)
BamA E470K	pMS 1840	BamA plasmid (pMS 1224) with BamA E470K point mutation	This study
BAM (BamA E470K)	pMS 1841	WT BAM plasmid with BamA gene with E470K point mutation	This study
BamA-His E470K	pMS 1847	WT BAM plasmid with an N-terminally 6-His tagged BamA gene with E470K point mutation, and deletion of BamBCDE lipoproteins	This study
BamADE-His	pMS 1848	WT BamABCDE plasmid with deletion of BamBC lipoproteins	This study
BamAD-His	pMS 1849	WT BamABCDE plasmid with a C-terminally 8-His tagged BamD gene, and deletion of BamBCE lipoproteins	This study
BamAB-His	pMS 1851	WT BamABCDE plasmid with a C-terminally 8-His tagged BamB gene, and a deletion of BamCDE lipoproteins	This study

Table S2. Oligonucleotides used in this study

Oligonucleotide	Sequence (5' → 3')	Description
U703	GATATACCATGGCTTGCCCGA	Upper primer for site-directed mutagenesis to introduce an N-terminal cysteine into the tOmpA gene
L703	GGTGTTATCTTTCGGGCAAGC	Lower primer for site-directed mutagenesis to introduce an N-terminal cysteine into the tOmpA gene
BamA ∆POTRA 1 gene fragment	cgtctcactggtgaaaagaaaaaccaccctggcgcccaatacgcaaaccgcctctcc<br>ccgcgcgttggccgattcattaatgcagctggcacgacaggtttcccgactggaaagcg<br>ggcagtgagcgcaacgcaattaatgtgagttagcgcgaattgatctggtttgacagctta<br>tcatcgactgcacggtgcaccaatgcttctggcgtcaggcagccatcggaagctgtggt<br>atggctgtgcaggtcgtaaatcactgcataattcgtgtcgctcaaggcgcactcccgttct<br>ggataatgttttttgcgccgacatcataacggttctggcaaatattctgaaatgagctgttg<br>acaattaatcatccggctcgtataatgtgtggaattgtgagcggataacaatttcacacag<br>gaaacagcaTATGGTTAGGAAGAACGCATAATAACGATGGCGAT<br>GAAAAAGTTGCTCATAGCGTCGCTGCTGTTTAGCAGCGCCA<br>CCGTATACGGTGCTGAAGGGTTCGTACCGACCATTGCCAGC<br>ATTACTTTCTCCGGTAACAAATCGGTGAAAGATGACATGCTG<br>AAGCAAAACCTCGAGGCTTCTGGTGTGCGTGTGGGCGAAT<br>CCCTCGATCGCACCACCATTGCCGATATCGAGAAAGGTCTG<br>GAAGACTTCTACTACAGCGTCGGTAAATATAGCGCCAGCGTA<br>AAAGCTGTCGTGACCCCGCTGCCGCGCAACCGTGTTGACC<br>TAAAACTGGTGTTCCAGGAAGGTGTGTCAGCTGAAATCCAG<br>CAAATTAACATTGTTGGTAACCATGCTTTCACCACCGACGAA<br>CTGATCTCTCATTTCCAACTGCGTGACGAAGTGCCGTGGTG<br>GAACGTGGTAGGCGATCGTAAATACCAGAAACAGAAACTGG<br>CGGGCGACCTTGAAACCCTGCGCAGCTACTATCTGGATCGC<br>GGTTATGCCCGTTTCAACATCGACTCTACCCAGGTCAGTCT<br>GACGCCAGATAAAAAAGGTATTTACGTCACGGTGAACATCAC<br>CGAAGGCGATCAGTACAAGCTTTCTGGCGTTGAAGTGAGC<br>GGCAACCTTGCCGGGCACTCCGCTGAAATTGAGCAGCTGA<br>CTAAGATCGAGCCGGGTGAGCTGTATAACGGCACCAAAGTG<br>ACCAAGATGGAAGATGACATCAAAAAGCTT	Gene fragment used in Gibson assembly reaction to substitute wildtype BamA gene with a BamA gene missing POTRA1 domain
BamA ∆POTRA 1-2 gene fragment	cgtctcactggtgaaaagaaaaaccaccctggcgcccaatacgcaaaccgcctctcc<br>ccgcgcgttggccgattcattaatgcagctggcacgacaggtttcccgactggaaagcg<br>ggcagtgagcgcaacgcaattaatgtgagttagcgcgaattgatctggtttgacagctta<br>tcatcgactgcacggtgcaccaatgcttctggcgtcaggcagccatcggaagctgtggt<br>atggctgtgcaggtcgtaaatcactgcataattcgtgtcgctcaaggcgcactcccgttct<br>ggataatgttttttgcgccgacatcataacggttctggcaaatattctgaaatgagctgttg<br>acaattaatcatccggctcgtataatgtgtggaattgtgagcggataacaatttcacacag<br>gaaacagcaTATGGTTAGGAAGAACGCATAATAACGATGGCGAT<br>GAAAAAGTTGCTCATAGCGTCGCTGCTGTTTAGCAGCGCCA<br>CCGTATACGGTGCTGAAGGGTTCGTAGGTGTGTCAGCTGAA	Gene fragment used in Gibson assembly reaction to substitute wildtype BamA gene with a BamA gene missing

	ATCCAGCAAATTAACATTGTTGGTAACCATGCTTTCACCACC<br>GACGAACTGATCTCTCATTTCCAACTGCGTGACGAAGTGCC<br>GTGGTGGAACGTGGTAGGCGATCGTAAATACCAGAAACAGA<br>AACTGGCGGGCGACCTTGAAACCCTGCGCAGCTACTATCTG<br>GATCGCGGTTATGCCCGTTTCAACATCGACTCTACCCAGGT<br>CAGTCTGACGCCAGATAAAAAAGGTATTTACGTCACGGTGA<br>ACATCACCGAAGGCGATCAGTACAAGCTTTCTGGCGTTGAA<br>GTGAGCGGCAACCTTGCCGGGCACTCCGCTGAAATTGAGC<br>AGCTGACTAAGATCGAGCCGGGTGAGCTGTATAACGGCACC<br>AAAGTGACCAAGATGGAAGATGACATCAAAAAGCTT	POTRA1-2 domains
BamA ∆POTRA 1-3 gene fragment	cgtctcactggtgaaaagaaaaaccaccctggcgcccaatacgcaaaccgcctctcc<br>ccgcgcgttggccgattcattaatgcagctggcacgacaggtttcccgactggaaagcg<br>ggcagtgagcgcaacgcaattaatgtgagttagcgcgaattgatctggtttgacagctta<br>tcatcgactgcacggtgcaccaatgcttctggcgtcaggcagccatcggaagctgtggt<br>atggctgtgcaggtcgtaaatcactgcataattcgtgtcgctcaaggcgcactcccgttct<br>ggataatgttttttgcgccgacatcataacggttctggcaaatattctgaaatgagctgttg<br>acaattaatcatccggctcgtataatgtgtggaattgtgagcggataacaatttcacacag<br>gaaacagcaTATGGTTAGGAAGAACGCATAATAACGATGGCGAT<br>GAAAAAGTTGCTCATAGCGTCGCTGCTGTTTAGCAGCGCCA<br>CCGTATACGGTGCTGAAGGGTTCGTAACCGAAGGCGATCAG<br>TACAAGCTTTCTGGCGTTGAAGTGAGCGGCAACCTTGCCG<br>GGCACTCCGCTGAAATTGAGCAGCTGACTAAGATCGAGCC<br>GGGTGAGCTGTATAACGGCACCAAAGTGACCAAGATGGAA<br>GATGACATCAAAAAGCTT	Gene fragment used in Gibson assembly reaction to substitute wildtype BamA gene with a BamA gene missing POTRA1-3 domains
BamA-His gene fragment	CAATCCGCCCTCACTACAACCGcgtctcactggtgaaaagaaaaacca<br>ccctggcgcccaatacgcaaaccgcctctccccgcgcgttggccgattcattaatgcag<br>ctggcacgacaggtttcccgactggaaagcgggcagtgagcgcaacgcaattaatgt<br>gagttagcgcgaattgatctggtttgacagcttatcatcgactgcacggtgcaccaatgct<br>tctggcgtcaggcagccatcggaagctgtggtatggctgtgcaggtcgtaaatcactgc<br>ataattcgtgtcgctcaaggcgcactcccgttctggataatgttttttgcgccgacatcataa<br>cggttctggcaaatattctgaaatgagctgttgacaattaatcatccggctcgtataatgtgt<br>ggaattgtgagcggataacaatttcacacaggaaacagcaTATGGTTAGGAA<br>GAACGCATAATAACGATGGCGATGAAAAAGTTGCTCATAGCG<br>TCGCTGCTGTTTAGCAGCGCCACCGTATACGGTGCTCACCA<br>TCACCATCACCATGCTGCAGAAGGGTTCGTAGTGAAAGATAT<br>TCATTTCGAAGGCCTTCAGCGTGTCGCCGTTGGTGCGGCC<br>CTCCTCAGTATGCCGGTGCGCACAGGCGACACGGTTAATGA<br>TGAAGATATCAGTAATACCATTCGCGCTCTGTTTGCTACCGG<br>CAACTTTGAGGATGTTCGCGTCCTTCGTGATGGTGATACCC<br>TTCTGGTTCAGGTAAAAGAACGTCCGACCATTGCCAGCATT<br>ACTTTCTCCGGTAACAAATCGGTGAAAGATGACATGCTGAA<br>GCAAAACCTCGAGGCTTCTGGTGTGCGTGTGGGCGAATCC<br>CTCGATCGCACCACCATTGCCGATATCGAGAAAGGTCTGGA<br>AGACTTCTACTACAGCGTCGGTAAATATAGCGCCAGCGTAAA<br>AGCTGTCGTGACCCCGCTGCCGCGCAACCGTGTTGACCTA<br>AAACTGGTGTTCCAGGAAGGTGTGTCAGCTGAAATCCAGCA<br>AATTAACATTGTTGGTAACCATGCTTTCACCACCGACGAACT<br>GATCTCTCATTTCCAACTGCGTGACGAAGTGCCGTGGTGGA<br>ACGTGGTAGGCGATCGTAAATACCAGAAACAGAAACTGGCG<br>GGCGACCTTGAAACCCTGCGCAGCTACTATCTGGATCGCG<br>GTTATGCCCGTTTCAACATCGACTCTACCCAGGTCAGTCTG<br>ACGCCAGATAAAAAAGGTATTTACGTCACGGTGAACATCACC<br>GAAGGCGATCAGTACAAGCTTTCTGGCGTTGAAGTGAGCG<br>GCAACCTTGCCGGGCACTCCGCTGAAATTGAGCAGCTGAC<br>TAAGATCGAGCCGGGTGAGCTGTATAACGGCACCAAAGTGA<br>CCAAGATGGAAGATGACATCAAAAAGCTTCTCGGTCGCTAT	Gene fragment containing an N-terminal 6-His tagged BamA flanked at the end by an XbaI site to exclude BamBCDE when used with restriction cloning

	GGTTATGCCTATCCGCGCGTACAGTCGATGCCCGAAATTAA CGATGCCGACAAAACCGTTAAATTACGTGTGAACGTTGATG CGGGTAACCGTTTCTACGTGCGTAAGATCCGTTTTGAAGGT AACGATACCTCGAAAGATGCCGTCCTGCGTCGCGAAATGCG TCAGATGGAAGGTGCATGGCTGGGGAGCGATCTGGTCGAT CAGGGTAAGGAGCGTCTGAATCGTCTGGGCTTCTTTGAAAC TGTCGATACCGATACCCAACGTGTTCCGGGTAGCCCGGACC AGGTTGATGTCGTCTACAAGGTAAAAGAGCGCAACACCGGT AGCTTCAACTTTGGTATTGGTTACGGTACTGAAAGTGGCGT GAGCTTCCAGGCTGGTGTGCAGCAGGATAACTGGTTAGGTA CAGGTTATGCTGTTGGTATCAACGGGACCAAAAACGATTACC AGACCTATGCTGAACTGTCGGTAACCAACCCGTACTTCACC GTAGATGGCGTAAGCCTCGGTGGTCGTCTCTTCTATAATGAC TTCCAGGCAGATGACGCCGACCTGTCCGACTATACCAACAA GAGTTATGGTACAGACGTGACGTTGGGCTTCCCGATTAACG AATATAACTCGCTGCGTGCAGGTCTGGGTTATGTACATAACT CCCTGTCCAACATGCAGCCTCAGGTTGCGATGTGGCGTTAT CTGTACTCTATGGGTGAACATCCGAGCACCTCTGATCAGGA TAACAGCTTCAAAACGGACGACTTCACGTTCAACTATGGTTG GACCTATAACAAGCTTGACCGTGGTTACTTCCCGACAGATG GTTCACGTGTCAACCTGACCGGTAAAGTGACCATTCCTGGA TCGGATAACGAATACTACAAAGTGACGTTAGACACGGCGAC TTATGTGCCGATCGATGACGATCACAAATGGGTTGTTCTGG GGCGTACCCGCTGGGGTTATGGTGATGGTTTAGGCGGCAA AGAGATGCCGTTCTACGAGAACTTCTATGCCGGTGGTTCCA GCACCGTGCGTGGCTTCCAGTCCAATACCATTGGTCCGAAA GCAGTTTACTTCCCGCATCAGGCCAGTAATTATGATCCGGAC TATGATTACGAATGTGCGACTCAGGACGGCGCGAAAGACCT GTGTAAATCGGATGATGCTGTAGGCGGTAACGCCATGGCGG TTGCCAGCCTCGAGTTCATCACCCCGACGCCGTTTATTAGC GATAAGTATGCTAACTCGGTTCGTACTTCCTTCTTCTGGGAT ATGGGTACCGTTTGGGATACAAACTGGGATTCCAGCCAATAT TCTGGATATCCGGACTATAGTGATCCAAGCAATATCCGTATGT CTGCGGGTATCGCATTACAATGGATGTCCCCATTGGGGCCG TTGGTGTTCTCCTACGCCCAGCCGTTCAAAAAGTACGATGG AGACAAGGCAGAACAGTTCCAGTTTAACATCGGTAAAACCT GGTAAGTGGGATCTGAGAGGGACCCGtctagaATGCAATTGC GTAAATTACT	
U2804	TATGCTaaaCTGTCGGTAACCAACCCGTACTTCACCGT	Upper primer for site-directed mutagenesis for production of pMS 1840
L2804	CGACAGtttAGCATAGGTCTGGTAATCGTTTTTGGTCCCGTTG A	Lower primer for site-directed mutagenesis for production of pMS 1840
U2805	GCAATAGACAGGGACCCTCGGATCTTAGGGAGATTTGGGATCTTGA	Upper primer for amplification of BamDE for addition after BamA (and remove BCDE) WT BAM plasmid

L2805	gcaggtcgactctagagGATCTTAGTGG	Lower primer for amplification of BamDE for addition after BamA (and remove BCDE) WT BAM plasmid
BamD-His gene fragment	GGGACCCTCGGATCTTAGGGAGATTTGGGATCTTGAGGAAAGTCAAA<br>ACGTCATGACGCGCATGAAATATCTGGTGGCAGCCGCCACACTAAG<br>CCTGTTTTTGGCGGGTTGCTCGGGGTCAAAGGAAGAAGTACCTGATA<br>ATCCGCCAAATGAAATTTACGCGACTGCACAACAAAAGCTGCAGGA<br>CGGTAACTGGAGACAGGCAATAACGCAACTGGAAGCGTTAGATAATC<br>GCTATCCGTTTGGTCCGTATTCGCAGCAGGTGCAGCTGGATCTCATC<br>TACGCCTACTATAAAAACGCCGATTTGCCGTTAGCACAGGCTGCCAT<br>CGATCGTTTTATTCGCCTTAACCCGACCCATCCGAATATCGATTATGTC<br>ATGTACATGCGTGGCCTGACCAATATGGCGCTGGATGACAGTGCGCT<br>GCAAGGGTTCTTTGGCGTCGATCGTAGCGATCGCGATCCTCAACATG<br>CACGAGCTGCGTTTAGTGACTTTTCCAAACTGGTGCGCGGCTATCCG<br>AACAGTCAGTACACCACCGATGCCACCAAACGTCTGGTATTCCTGAA<br>AGATCGTCTGGCGAAATATGAATACTCCGTGGCCGAGTACTATACAGA<br>ACGTGGCGCATGGGTTGCCGTCGTTAACCGCGTAGAAGGCATGTTG<br>CGCGACTACCCGGATACCCAGGCTACGCGTGATGCGCTGCCGCTG<br>ATGGAAAATGCATACCGTCAGATGCAGATGAATGCGCAAGCTGAAAA<br>AGTAGCGAAAATCATCGCCGCAAACAGCAGCAATACAgcggccGCAC<br>TCGAGCACCACCACCACCACCACCACCACTAAtctaga	Gene fragment containing an C-terminal 8-His tagged BamD flanked at the end by an XbaI site to exclude BamBCDE when used with restriction cloning
BamAB-His gene fragment	TTAGGAAGAACGCATAATAACcATGGCGATGAAAAAGTTGCTCATAGC<br>GTCGCTGCTGTTTAGCAGCGCCACCGTATACGGTGCTGAAGGGTTC<br>GTAGTGAAAGATATTCATTTCGAAGGCCTTCAGCGTGTCGCCGTTGGT<br>GCGGCCCTCCTCAGTATGCCGGTGCGCACAGGCGACACGGTTAAT<br>GATGAAGATATCAGTAATACCATTCGCGCTCTGTTTGCTACCGGCAAC<br>TTTGAGGATGTTCGCGTCCTTCGTGATGGTGATACCCTTCTGGTTCAG<br>GTAAAAGAACGTCCGACCATTGCCAGCATTACTTTCTCCGGTAACAA<br>ATCGGTGAAAGATGACATGCTGAAGCAAAACCTCGAGGCTTCTGGTG<br>TGCGTGTGGGCGAATCCCTCGATCGCACCACCATTGCCGATATCGA<br>GAAAGGTCTGGAAGACTTCTACTACAGCGTCGGTAAATATAGCGCCA<br>GCGTAAAAGCTGTCGTGACCCCGCTGCCGCGCAACCGTGTTGACC<br>TAAAACTGGTGTTCCAGGAAGGTGTGTCAGCTGAAATCCAGCAAATTA<br>ACATTGTTGGTAACCATGCTTTCACCACCGACGAACTGATCTCTCATTT<br>CCAACTGCGTGACGAAGTGCCGTGGTGGAACGTGGTAGGCGATCGT<br>AAATACCAGAAACAGAAACTGGCGGGCGACCTTGAAACCCTGCGC<br>AGCTACTATCTGGATCGCGGTTATGCCCGTTTCAACATCGACTCTACC<br>CAGGTCAGTCTGACGCCAGATAAAAAAGGTATTTACGTCACGGTGAA<br>CATCACCGAAGGCGATCAGTACAAGCTTTCTGGCGTTGAAGTGAGC<br>GGCAACCTTGCCGGGCACTCCGCTGAAATTGAGCAGCTGACTAAGA<br>TCGAGCCGGGTGAGCTGTATAACGGCACCAAAGTGACCAAGATGGA<br>AGATGACATCAAAAAGCTTCTCGGTCGCTATGGTTATGCCTATCCGCG<br>CGTACAGTCGATGCCCGAAATTAACGATGCCGACAAAACCGTTAAAT<br>TACGTGTGAACGTTGATGCGGGTAACCGTTTCTACGTGCGTAAGATCC<br>GTTTTGAAGGTAACGATACCTCGAAAGATGCCGTCCTGCGTCGCGAA<br>ATGCGTCAGATGGAAGGTGCATGGCTGGGGAGCGATCTGGTCGATC<br>AGGGTAAGGAGCGTCTGAATCGTCTGGGCTTCTTTGAAACTGTCGATA<br>CCGATACCCAACGTGTTCCGGGTAGCCCGGACCAGGTTGATGTCGT	Gene fragment containing an C-terminal 8-His tagged BamB flanked at the end by an XbaI site to exclude BamBCDE when used with restriction cloning

	CTACAAGGTAAAAGAGCGCAACACCGGTAGCTTCAACTTTGGTATTG<br>GTTACGGTACTGAAAGTGGCGTGAGCTTCCAGGCTGGTGTGCAGCA<br>GGATAACTGGTTAGGTACAGGTTATGCTGTTGGTATCAACGGGACCAA<br>AAACGATTACCAGACCTATGCTGAACTGTCGGTAACCAACCCGTACT<br>TCACCGTAGATGGCGTAAGCCTCGGTGGTCGTCTCTTCTATAATGACT<br>TCCAGGCAGATGACGCCGACCTGTCCGACTATACCAACAAGAGTTAT<br>GGTACAGACGTGACGTTGGGCTTCCCGATTAACGAATATAACTCGCT<br>GCGTGCAGGTCTGGGTTATGTACATAACTCCCTGTCCAACATGCAGC<br>CTCAGGTTGCGATGTGGCGTTATCTGTACTCTATGGGTGAACATCCGA<br>GCACCTCTGATCAGGATAACAGCTTCAAAACGGACGACTTCACGTTC<br>AACTATGGTTGGACCTATAACAAGCTTGACCGTGGTTACTTCCCGACA<br>GATGGTTCACGTGTCAACCTGACCGGTAAAGTGACCATTCCTGGATC<br>GGATAACGAATACTACAAAGTGACGTTAGACACGGCGACTTATGTGC<br>CGATCGATGACGATCACAAATGGGTTGTTCTGGGGCGTACCCGCTG<br>GGGTTATGGTGATGGTTTAGGCGGCAAAGAGATGCCGTTCTACGAGA<br>ACTTCTATGCCGGTGGTTCCAGCACCGTGCGTGGCTTCCAGTCCAAT<br>ACCATTGGTCCGAAAGCAGTTTACTTCCCGCATCAGGCCAGTAATTAT<br>GATCCGGACTATGATTACGAATGTGCGACTCAGGACGGCGCGAAAG<br>ACCTGTGTAAATCGGATGATGCTGTAGGCGGTAACGCgATGGCGGTT<br>GCCAGCCTCGAGTTCATCACCCCGACGCCGTTTATTAGCGATAAGTA<br>TGCTAACTCGGTTCGTACTTCCTTCTTCTGGGATATGGGTACCGTTTGG<br>GATACAAACTGGGATTCCAGCCAATATTCTGGATATCCGGACTATAGT<br>GATCCAAGCAATATCCGTATGTCTGCGGGTATCGCATTACAATGGATG<br>TCCCCATTGGGGCCGTTGGTGTTCTCCTACGCCCAGCCGTTCAAAA<br>AGTACGATGGAGACAAGGCAGAACAGTTCCAGTTTAACATCGGTAAA<br>ACCTGGTAAGGGACCCAGAAGGAGATATCATATGCAATTGCGTAAATT<br>ACTGCTGCCAGGACTGCTTTCCGTTACCCTTTTAAGCGGCTGTTCGC<br>TGTTTAACAGCGAAGAAGATGTGGTAAAGATGTCCCCATTGCCAACC<br>GTTGAAAACCAGTTTACGCCGACCACGGCGTGGAGCACTTCCGTTG<br>GTAGCGGCATTGGCAACTTCTATTCCAATCTTCATCCGGCACTGGCG<br>GACAACGTTGTCTATGCAGCGGACCGCGCTGGTTTAGTAAAAGCGC<br>TGAATGCGGATGATGGCAAAGAAATCTGGTCTGTCAGCCTGGCCGA<br>GAAAGATGGCTGGTTCTCTAAAGAGCCTGCATTACTTTCTGGCGGTGT<br>GACCGTGTCTGGTGGGCATGTCTACATTGGCAGCGAAAAGGCGCAG<br>GTTTACGCGCTGAATACCAGCGATGGTACTGTGGCATGGCAAACTAA<br>AGTCGCGGGTGAAGCACTTTCGCGCCCGGTGGTCAGCGACGGTCT<br>GGTGTTAATCCACACCAGTAACGGTCAGTTACAAGCGCTGAACGAAG<br>CTGACGGCGCTGTCAAATGGACAGTTAACCTCGATATGCCTTCGCTC<br>TCTTTGCGTGGCGAGTCTGCGCCGACAACGGCTTTTGGTGCGGCC<br>GTCGTGGGGGGCGATAATGGTCGCGTCAGCGCAGTGCTGATGGAA<br>CAGGGCCAGATGATTTGGCAGCAGCGTATTTCCCAGGCGACCGGTT<br>CTACCGAAATTGACCGTCTGAGCGATGTTGACACGACTCCCGTCGTT<br>GTTAACGGCGTTGTTTTCGCGCTGGCCTATAATGGTAACCTGACGGC<br>GCTTGATCTGCGCAGTGGTCAGATTATGTGGAAACGCGAACTGGGTT<br>CGGTGAATGATTTCATCgttGACGGCAATCGCATCTATCTGGTCGATCA<br>AAATGACCGGGTGATGGCGTTGACCATTGATGGCGGCGTTACGCTGT<br>GGACACAAAGCGATCTGCTGCATCGCCTGCTGACTTCTCCGGTGCT<br>GTATAATGGCAACCTGGTGGTCGGTGACAGTGAAGGTTATCTGCACT<br>GGATTAACGTCGAAGATGGTCGTTTCGTTGCCCAGCAAAAAGTTGATA<br>GTTCCGGTTTCCAGACTGAACCGGTTGCCGCTGACGGCAAACTGCT<br>GATCCAGGCAAAAGACGGAACCGTGTACTCTATTACACGTgcggccGC<br>ACTCGAGCACCACCACCACCACCACCACCACTAAgAGCTCtctagagt<br>cgacctgcaggcatgcaa	

SI References

1. E. J. Danoff, K. G. Fleming, Novel Kinetic Intermediates Populated along the Folding Pathway of the Transmembrane β-Barrel OmpA. *Biochemistry* **56**, 47-60 (2017).
2. V. Chaptal *et al.*, Quantification of Detergents Complexed with Membrane Proteins. *Scientific Reports* **7** (2017).
3. C. Prince, Z. Jia, Measurement of detergent concentration using 2,6-dimethylphenol in membrane-protein crystallization. *Acta Crystallographica Section D Biological Crystallography* **68**, 1694-1696 (2012).
4. M. Botte *et al.*, A central cavity within the holo-translocon suggests a mechanism for membrane protein insertion. *Scientific Reports* **6** (2016).
5. Y. Tanaka *et al.*, 2.8-Å crystal structure of Escherichia coli YidC revealing all core regions, including flexible C2 loop. *Biochemical and Biophysical Research Communications* **505**, 141-145 (2018).
6. A. Furukawa *et al.*, Tunnel Formation Inferred from the I-Form Structures of the Proton-Driven Protein Secretion Motor SecDF. *Cell Reports* **19**, 895-901 (2017).
7. D. R. Weaver, D. N. Amin, G. M. King, The conformations and basal conformational dynamics of translocation factor SecDF vary with translocon SecYEG interaction. *Journal of Biological Chemistry* **298** (2022).
8. P. White *et al.*, The role of membrane destabilisation and protein dynamics in BAM catalysed OMP folding. *Nature Communications* **12** (2021).
9. J. A. Hobot, E. Carlemalm, W. Villiger, E. Kellenberger, Periplasmic gel: new concept resulting from the reinvestigation of bacterial cell envelope ultrastructure by new methods. *Journal of Bacteriology* **160**, 143-152 (1984).
10. L. L. Graham, R. Harris, W. Villiger, T. J. Beveridge, Freeze-substitution of gram-negative eubacteria: general cell morphology and envelope profiles. *Journal of Bacteriology* **173**, 1623-1633 (1991).
11. R. F. Collins *et al.*, The 3D structure of a periplasm-spanning platform required for assembly of group 1 capsular polysaccharides in Escherichia coli. *Proceedings of the National Academy of Sciences* **104**, 2390-2395 (2007).
12. T. C. Marlovits *et al.*, Structural Insights into the Assembly of the Type III Secretion Needle Complex. *Science* **306**, 1040-1042 (2004).
13. V. r. R. F. Matias, A. Al-Amoudi, J. Dubochet, T. J. Beveridge, Cryo-Transmission Electron Microscopy of Frozen-Hydrated Sections of Escherichia coli and Pseudomonas aeruginosa. *Journal of Bacteriology* **185**, 6112-6118 (2003).
14. G. Roman-Hernandez, J. H. Peterson, H. D. Bernstein, Reconstitution of bacterial autotransporter assembly using purified components. *eLife* **3** (2014).
15. L. R. Warner, P. Z. Gatzeva-Topalova, P. A. Doerner, A. Pardi, M. C. Sousa, Flexibility in the Periplasmic Domain of BamA Is Important for Function. *Structure* **25**, 94-106 (2017).
16. P. A. Doerner, M. C. Sousa, Extreme Dynamics in the BamA β-Barrel Seam. *Biochemistry* **56**, 3142-3149 (2017).
